# Initial Characterization of the Chloroplast Genome of *Vicia sepium*, an Important Wild Resource Plant, and Related Inferences About Its Evolution

**DOI:** 10.3389/fgene.2020.00073

**Published:** 2020-02-20

**Authors:** Chaoyang Li, Yunlin Zhao, Zhenggang Xu, Guiyan Yang, Jiao Peng, Xiaoyun Peng

**Affiliations:** ^1^ Hunan Research Center of Engineering Technology for Utilization of Environmental and Resources Plant, Central South University of Forestry and Technology, Changsha, China; ^2^ Hunan Urban and Rural Ecological Planning and Restoration Engineering Research Center, Hunan City University, Yiyang, China

**Keywords:** chloroplast genome, comparative analysis, phylogenetic analysis, positive selection, *Vicia sepium*

## Abstract

Lack of complete genomic information concerning *Vicia sepium* (Fabaceae: Fabeae) precludes investigations of evolution and populational diversity of this perennial high-protein forage plant suitable for cultivation in extreme conditions. Here, we present the complete and annotated chloroplast genome of this important wild resource plant. *V. sepium* chloroplast genome includes 76 protein-coding genes, 29 tRNA genes, 4 rRNA genes, and 1 pseudogene. Its 124,095 bp sequence has a loss of one inverted repeat (IR). The GC content of the whole genome, the protein-coding, intron, tRNA, rRNA, and intergenic spacer regions was 35.0%, 36.7%, 34.6%, 52.3%, 54.2%, and 29.2%, respectively. Comparative analyses with plastids from related genera belonging to Fabeae demonstrated that the greatest variation in the *V. sepium* genome length occurred in protein-coding regions. In these regions, some genes and introns were lost or gained; for example, *ycf4*, *clpP* intron, and *rpl16* intron deletions and *rpl20* and *ORF292* insertions were observed. Twelve highly divergent regions, 66 simple sequence repeats (SSRs) and 27 repeat sequences were also found in these regions. Detailed evolutionary rate analysis of protein-coding genes showed that *Vicia* species exhibit additional interesting characteristics including positive selection of *ccsA*, *clpP*, *rpl32*, *rpl33*, *rpoC1*, *rps15*, *rps2, rps4*, and *rps7*, and the evolutionary rates of *atpA*, *accD*, and *rps2* in *Vicia* are significantly accelerated. These genes are important candidate genes for understanding the evolutionary strategies of *Vicia* and other genera in Fabeae. The phylogenetic analysis showed that *Vicia* and *Lens* are included in the same clade and that *Vicia* is paraphyletic. These results provide evidence regarding the evolutionary history of the chloroplast genome.

## Introduction

Complete chloroplast sequences are indispensable for analyzing genome evolution and phylogenetics (Sabir et al., 2014; Moner et al., 2018). These sequences offer two advantages over genomic ones, namely, a high degree of conservation and a relatively compact gene alignment, resulting from symbiotic horizontal transfer (Timmis et al., 2004). In angiosperms, the chloroplast is a uniparentally inherited organelle. It originated from a cyanobacterium-like organism through an endosymbiosis event. Compared to the nuclear genome, chloroplast genomes, with a quadripartite circular structure, exhibit highly conserved sizes, structures and gene contents across photosynthetic plants (Wicke et al., 2011). Nuclear genomes are highly complex because of the high frequency of the loss and gain of genetic material at any time (Wolfe et al., 1987), making the identification of orthologous genes difficult. Evolutionary and phylogenetic analyses based on complete chloroplast sequences can provide more valuable information of a higher quality than that obtained by analysis of one or more gene loci (Martin et al., 2005). Complete chloroplast sequence datasets contain all site patterns (or all genes) for the reconstruction of evolutionary history. The comparison of complete genomes can reduce the sampling error inherent in analyses of only one or a few genes. That is not to say that we oppose the use of one or a few genes in evolutionary studies, but we instead suggest the investigation of conflicts between complete chloroplast genomes and analyses of one or a few genes that may indicate crucial evolutionary events. Another advantage of the chloroplast genome is that it contributes to structural diversity at low taxonomic levels and among basal lineages. Although genome organization is relatively well conserved in angiosperms, several types of structural diversity have been found. This structural diversity, including the loss of one copy of IRs, gene and intron gains or losses, large inversions, expansions, contractions and localized hypermutable phenomena, provides a powerful tool for evaluating genomic evolutionary history. For example, the loss of one IR is observed in the inverted-repeat-lacking clade (IRLC) (Sabir et al., 2014); the loss of *accD*, *psaI*, *ycf4*, *rpl33*, *clpP*, and *rps16* resulting in gene function loss is observed in various legume lineages; a 36-kb inversion is observed in the *Genistoid* clade; a 39-kb inversion is observed in *Robinia* (Keller et al., 2017); and hypermutation of *ycf4* is observed in *Lathyrus* (Magee et al., 2010). With the development of high-throughput sequencing, more than 800 complete chloroplast genomes have been made available in the National Center for Biotechnology Information (NCBI) database (Asaf et al., 2017a).

The Fabaceae family, especially the Papilionoideae subfamily, is considered a model system for understanding the mechanisms of chloroplast genome evolution due to the presence of major genome rearrangements in this group such as loss of one IR, gene and intron gains and losses, large inversions, expansions, contractions and localized hypermutable regions (Sabir et al., 2014; Keller et al., 2017). However, the mechanisms of these chloroplast genome rearrangements are not known (Sveinsson and Cronk, 2016). Some scholars believe that these genome rearrangements within the Fabaceae chloroplast genomes may be derived from the loss of one copy of IRs; however, *Medicago* and *Cicer* species, which exhibit the typical conserved quadripartite structure found in angiosperms (Jansen et al., 2005), also present extensive chloroplast genome rearrangements (Jansen et al., 2008; Sveinsson and Cronk, 2016). Therefore, further in-depth research on the mechanisms of chloroplast genome evolution is needed.

Previous research on Fabaceae chloroplast genomes demonstrated that the deletion or addition of genes and introns, inversions, repeats, and nucleotide variability can result in significant changes in genome length, GC content, and gene composition and orientation (Lei et al., 2016; Yin et al., 2017; Wang et al., 2018). In these genomes, coding regions are better conserved than intergenic spacer (IGS) regions (Sabir et al., 2014; Asaf et al., 2017b; Yin et al., 2018). However, it is unclear whether a consistent pattern in the genomic variation can be observed in species of the tribe Fabeae, which belong to Fabaceae. A possible explanation for these results may be the lack of complete genomic information for Fabeae. To date, 21 complete Fabeae chloroplast genomes have been sequenced (including 18 in the last four years), mainly from the genus *Lathyrus* (13) and a few from the genera *Lens* (1), *Pisum* (4) and *Vicia* (3). Another possible explanation is the structural diversity among Papilionoideae (Jansen et al., 2008; Sabir et al., 2014; Sveinsson and Cronk, 2016). For example, even within the same genus, the *Trifolium subterraneum* (Fabaceae) chloroplast genome exhibits 14-18 inversions, while there are only 3 inversions in *Trifolium grandiflorum* and *Trifolium aureum* (Sabir et al., 2014). Therefore, the study of the genomic variation and phylogeny of Fabeae species can provide a basis for understanding chloroplast genome evolution.

*Vicia sepium* (Bush vetch), belonging to the tribe Fabeae, is an important wild resource plant with a wide distribution area (Maxted, 1995), various ﬂowering periods from May to November, abundant proteins, and suitability for cultivation in extreme cold and dry conditions (Maršalkienė, 2016) and can be used as a good potential perennial forage. Additionally, compared with other legumes, *V. sepium* provides herbage for a long period because of its perennial habit (Maršalkienė, 2016). This plant also produces extrafloral nectaries to attract ants, which act as plant defenders by preying on arthropod herbivores or interrupting their oviposition or feeding (Lenoir and Pihlgren, 2006). However, previous studies on *V. sepium* have mainly focused on the morphological characteristics (Maršalkienė, 2016) and classification (Schaefer et al., 2012; Jaaska, 2015) of this plant and the relationship between plants and insects (Kruess and Tscharntke, 2000; Lenoir and Pihlgren, 2006). Therefore, little is known regarding the nutrient content, genetic resources, and forage value of this species. As a result, no plant materials of *V. sepium* have been released for commercial production. However, another *Vicia* species, *Vicia sativa*, has been widely used as forage and for hay and silage production. A key difficulty in the use of *V. sativa* is the presence of a neurotoxic compound in its seeds (Huang et al., 2017). Therefore, the expansion of forage resources based on *Vicia* species is necessary.

Another difficulty in the utilization of *V. sepium* is that the taxonomy of some taxa in Fabeae remains controversial (Schaefer et al., 2012; Jaaska, 2015; Iberite et al., 2017) because of the high morphological variability among species. Notably, some variation in morphological characteristics is genetically fixed. For example, Iberite’s cultivation tests (Iberite et al., 2017) conducted in *V. sativa*, *Vicia barbazitae*, *Vicia grandiflora* and *V. sepium* showed that the characteristics of the leaf margins are maintained through successive generations. Recent molecular phylogenetic studies have focused on multitribe legumes or tribe level analyses of Fabeae (Schaefer et al., 2012). These studies have suggested that the taxonomy of some genera in Fabeae is not monophyletic. However, these phylogenetic studies did not use the complete chloroplast genome, instead using plastid DNA sequence data, such as the *matK*, *trnL*, *rbcL*, and nuclear ribosomal internal transcribed spacer (ITS) sequences. Therefore, it is necessary to acquire comprehensive knowledge regarding the organization and evolution of *V. sepium*.

Here, we present a new complete chloroplast genome of *V. sepium*, from the genus *Vicia*. We compare it with chloroplast genomes from related genera (*Lens*, *Pisum*, *Lathyrus*) belonging to tribe Fabeae. The aim of this work is to reveal the genome variation and phylogeny of Fabeae and the genus *Vicia* and to provide evidence regarding the history of chloroplast genome evolution.

## Materials and Methods

### Plant Material

The sample was collected from the Dongting Lake region (28°48′46.06″N, 112°21′10.19″E) and stored at the Hunan Research Center of Engineering Technology for Utilization of Environmental and Resources Plant, China, under accession number 20170707JJ. Plant sampling was performed in areas that were not privately owned or protected in any way, and no specific permits were required for this study. We collected mature *V. sepium* leaves and placed them in a liquid nitrogen container. Leaf samples were stored at -80°C until sequencing. Extraction of total chloroplast DNA was carried out with the Plant Chloroplast Purification Kit and Column Plant DNA Extraction Kit (Beijing Baiaolaibo Technology, Co., Ltd., China). The chloroplast DNA of *V. sepium* was fragmented using a Covaris M220 (Covaris, USA) instrument. Whole-genome sequencing and paired-end (PE) library construction were performed according to the method described by Zhang et al. (2017). Raw data were obtained through next-generation sequencing with PE 150-bp reads. Then, N-containing sequences and adapter sequences were removed. Sequences with a Q value less than 20 or an average four-base mass of less than 20 were also removed. Finally, if the length of the reads was less than 50 nt, the reads were removed. All the above filtering steps were performed using Trimmomatic v 0.32 (Bolger et al., 2014), and clean data for subsequent analysis were obtained. Then, all high-quality paired reads were assembled into contigs by using SOAPdenovo2 (Luo et al., 2012) and scaffolded by using SSPACE (Boetzer et al., 2011) to obtain the whole-genome sequence. In this process, different K-mers were selected first for assembly, and the best K-mer, *k*=25, was chosen to obtain the assemblies. The above K parameter was determined on the basis of a K-mer curve and experience. Finally, one contig of 124,095 bp was obtained.

### Genome Annotation and Sequence Architecture

Our previous study used the programs CpGAVAS (Liu et al., 2012) and DOGMA (Wyman et al., 2004) to annotate the complete chloroplast genome of *V. sepium* (Li et al., 2018). In this study, to study genomic evolution between *V. sepium* and its related species in Fabeae, the same *V. sepium* genome was annotated in Plann (Huang and Cronk, 2015) against the *V. sativa* genome (NC027155). Gene mapping and relative synonymous codon usage (RSCU) were performed in OGDRAW v1.2 (Lohse et al., 2013) and DAMBE6 (Xia, 2017) according to Dong’s method (Dong et al., 2019).

### SSRs and Repeated Sequences Analysis

We detected SSRs by referring to the method of Lei et al. (2016) using the MISA Perl Script (Thiel et al., 2003) with parameter settings of 8 for mono-, 4 for di- and tri-, and 3 for tetra-, penta- and hexa-nucleotide SSRs. Forward, palindromic, reverse, and complement sequences were identified as described by Cauz-Santos et al. (2017) using REPuter (Kurtz et al., 2001) with 90% or greater sequence identity and a length of 30 bp or longer. Tandem repeats were identified using Tandem Repeats Finder version 4.09 (Benson, 1999) with default parameters.

### Comparative Analysis

Blast ring image generator (BRIG) (Alikhan et al., 2011) and mVISTA (Frazer et al., 2004) software were used to compare the complete chloroplast genome variation in all available Fabeae chloroplast genomes using *V. sepium* annotation as a reference. BRIG focus on protein coding segment variation and mVISTA align whole chloroplast genome without discrimination. All the species were included the following twenty-one Fabeae species and one Cicereae species (*Cicer arietinum*), listed with the corresponding GenBank accession numbers: *V. sepium*, *V. sativa* (NC027155), *V. faba* (KF042344), *Pisum abyssinicum* (NC037830), *Pisum sativum* (NC014057), *Pisum sativum* subsp. Elatius (NC039371), *Pisum fulvum* (NC036828), *Lens culinaris* (NC027152), *Lathyrus pubescens* (NC027079), *Lathyrus venosus* (NC027080), *Lathyrus palustris* (NC027078), *Lathyrus japonicus* (NC027075), *Lathyrus ochroleucus* (NC027077), *Lathyrus davidii* (NC027073), *Lathyrus littoralis* (NC027076), *Lathyrus inconspicuus* (NC027149), *Lathyrus graminifolius* (NC027074), *Lathyrus tingitanus* (NC027151), *Lathyrus clymenum* (NC027148), *Lathyrus sativus* (NC014063), *Lathyrus odoratus* (NC027150), and *C. arietinum* (NC011163). Genome rearrangement relative to *V. sepium* was performed in Mauve (Darling et al., 2004).

### Phylogenetic Analysis

To determine the phylogenetic position of *V. sepium* within Fabeae, four datasets were used to construct the following phylogenetic trees for Fabeae: (I) the complete chloroplast genomes of 21 Fabeae species and *C. arietinum* (that is, the same 22 species in the comparative analysis); (II) the conserved chloroplast protein-coding sequences of 21 Fabeae species and *C. arietinum* (that is, the same 22 species in the comparative analysis); (III) the *rbcL* gene sequences of 50 Fabeae species, *Trifolium pretense* and *T. repens*; and (IV) the *matK* gene sequences of 62 Fabeae species, *T. pretense* and *T. repens*. The names of the species included in the four phylogenetic analyses can be found in **Table S1**.

Specifically, the conserved chloroplast protein-coding sequence of each species comprised 70 concatenated homologous genes shared among twenty-two related species. These genes were *atpA*, *atpB*, *atpE, atpF*, *atpH*, *atpI*, *ccsA*, *cemA*, *clpP*, *matK*, *ndhA*, *ndhB*, *ndhC*, *ndhD*, *ndhE*, *ndhF*, *ndhG*, *ndhH*, *ndhI*, *ndhJ*, *ndhK*, *petA*, *petB*, *petD*, *petG*, *petL*, *petN*, *psaA*, *psaB*, *psaC*, *psaJ*, *psbA*, *psbB*, *psbC*, *psbD*, *psbE*, *psbF*, *psbH*, *psbI*, *psbK*, *psbL*, *psbM*, *psbN*, *psbT*, *psbZ*, *rbcL*, *rpl14*, *rpl16*, *rpl2*, *rpl20*, *rpl23*, *rpl32*, *rpl33*, *rpl36*, *rpoA, rpoB*, *rpoC1*, *rpoC2*, *rps11*, *rps12*, *rps14*, *rps15*, *rps19*, *rps2*, *rps3*, *rps4*, *rps7*, *rps8*, *ycf1*, *ycf2*, and *ycf3*.

All datasets were aligned using MAFFT v7.380 (Katoh and Standley, 2013) under the FFT-NS-2 default setting. The alignments were used for phylogenetic analysis. All alignments were used to construct phylogenetic trees *via* the neighbor joining (NJ) method in MEGA7.0 (Kumar et al., 2016) under the default settings. Then, we obtained four NJ trees.

In addition, we used another method, the maximum likelihood (ML) method, to construct a phylogenetic tree based on conserved chloroplast protein-coding sequences. The aim of this work was to test the effects of different methods on the phylogenetic relationships of Fabeae species. First, we used MAFFT v7.380 to align twenty-two conserved chloroplast protein-coding sequences under the FFT-NS-2 default settings. Second, ModelTest was employed to find the best model in MEGA7.0. Finally, the tree was constructed using the ML method with the GTR+G+I model and 1,000 bootstrap replicates. *C. arietinum* was selected as the outgroup.

### Evolutionary Rate Analysis

To determine the sequence divergence of the complete chloroplast genomes, the average pairwise sequence distances of twenty-one Fabeae species and one Cicereae species (that is, the same 22 species in the comparative analysis) were calculated. After alignment with MAFFT v7.380, the average pairwise sequence distances (K2P rate) of these species were presented according to Asaf’s method using MEGA7 (Kimura, 1980; Asaf et al., 2017b).

Additionally, the synonymous (Ks) and nonsynonymous (Ka) nucleotide substitution rates as well as the Ka/Ks ratio were used to calculate the sequence divergence of other homologous protein-coding regions. All twenty-one available chloroplast genomes belonging to the genera *Vicia*, *Pisum*, *Lens*, and *Lathyrus* were selected for this analysis. These species were divided into two groups: (I) within *Vicia*: *V. sepium*, *V. sativa*, *V. faba*; (II) outside of *Vicia* (or other genera): *V. sepium*, *P. abyssinicum*, *P. sativum*, *P. sativum* subsp. Elatius, *P. fulvum*, *L. pubescens*, *L. venosus*, *L. palustris*, *L. japonicus*, *L. ochroleucus*, *L. davidii*, *L. littoralis*, *L. inconspicuus*, *L. graminifolius*, *L. tingitanus*, *L. clymenum*, *L. sativus*, and *L. odoratus*. A total of 71 homologous genes (**Table S2**) from these species were selected and examined separately. After aligning each gene using the ClustalW (Codons) program in MEGA7, the Ks, Ka, and Ka/Ks values between *V. sepium* and other species were determined according to Dong’s method (Dong et al., 2019) with the program from the PAML package (Yang and Nielsen, 1998). The two independent-samples t-test was used to examine the significance of the sequence divergence between *Vicia* and other genera. The *p*-values were determined with Levene's test. If the Levene's test result was less than 0.05, we used the unequal variance as the *p*-value; if not, we used the equal variance as the *p*-value.

Once *Vicia* showed a significantly higher Ka/Ks ratio than the other genera, codon-based likelihood analysis based on the branch model test in CodeML from the PAML package was carried out to identify the lineages in Fabeae that exhibited significantly high evolutionary rates. This test employed the user-defined topology of Fabeae lineages with five other lineages: A0 (*Cicer*), A1 (*Pisum* and *Lathyrus*), A2 (*Lens* and *Vicia*), A3 (*Lens*), and A4 (*Vicia*). This topology was constructed based on the concatenated DNA sequences of *matK* and *rbcL* (**Figure S1**) using the ML method with the GTR+G50 model in MEGA7.0. The method was the same as that used for the phylogenetic analysis described previously. A one-ratio model (model = 0) and a two-ratio model (model =2) were used to calculate the Ka/Ks ratio for each branch. A one-ratio model, or null model (model = 0), is one in which all clades (or all lineages) exhibit the same Ka/Ks ratio. A two-ratio model, or alternative model (model = 2), is one in which one or more clades present different Ka/Ks ratios. The transition/transversion and Ka/Ks ratios were set as automatically estimated. Codon frequencies were set as the F3 × 4 method. The hypotheses of the two-ratio model are described in **Table S3**. The likelihood ratio test (LRT) was used to find the best model (*P* < 0.05) through comparison of two different models. From the best model, we could infer whether a homologous gene showed accelerated evolution in *Vicia*. In addition, all genes exhibiting accelerated evolution were compared with two genes showing nonaccelerated evolution (*matK* and *rbcL*), in two ways. First, we compared their synonymous and nonsynonymous nucleotide substitution rates in Ks trees and Ka trees. The branch lengths representing the substitutions per synonymous site or nonsynonymous site were determined from the best model. Second, we compared their amino acid sequence differences. Amino acid sequence alignment was performed in Jalview v2.10.5 (Waterhouse et al., 2009).

## Results

### Chloroplast Genome Characteristics and Structure of *V. sepium*

**Figure 1:**
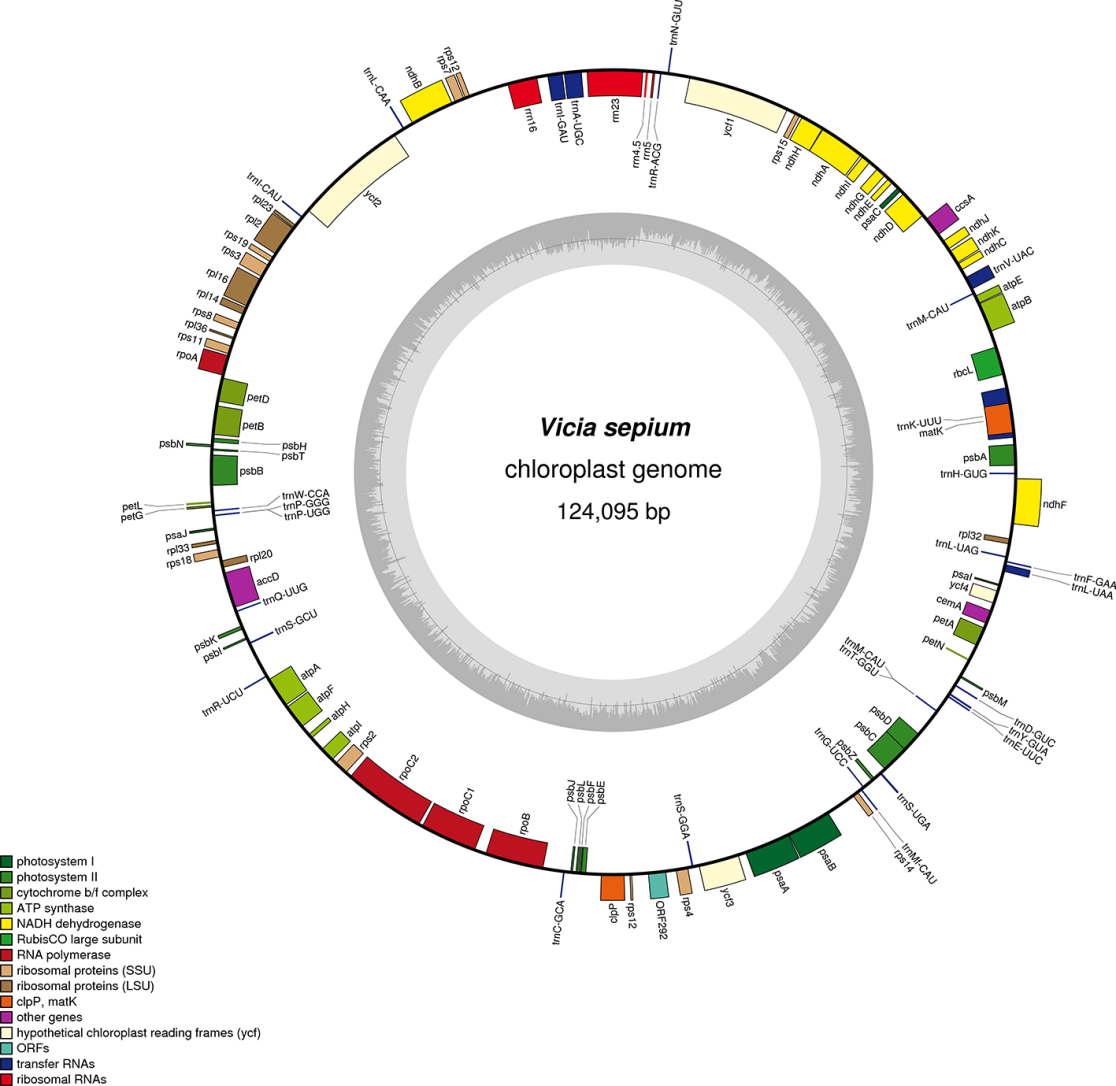
Gene map of the complete chloroplast genome of *V. sepium*. Genes inside the circle are transcribed clockwise, and those outside are transcribed counterclockwise. The different colors of the blocks represent different functional groups. The darker gray color of the inner circle corresponds to the GC content, and the lighter gray color corresponds to the AT content.

The original image data obtained by next-generation sequencing technology was converted into the original sequenced reads by CASAVA base calling analysis to obtain raw reads (10,808,365) or raw data (3.24 gigabytes). A total of 7,696,368 clean reads (2.31 gigabytes of clean data) with an average length of 150 bp were obtained after the adapter sequences and low-quality reads were removed. A single long contig of 124,095 bp was assembled using clean data *via de novo* assembly, forming a loop representing the whole chloroplast genome sequence of *V. sepium*. The *V. sepium* chloroplast genome, under GenBank accession number NC039595, showed the loss of one IR and contained 76 protein-coding genes, 29 tRNA genes, four rRNA genes and one pseudogene (*rpl23* Ψ). In particular, one unannotated protein-coding gene, *ORF292*, was identified (**Table 1**). The gene map of these 110 genes was presented (**Figure 1**). Among these protein-coding genes, 9 genes (*ndhA*, *ndhB*, *rpl2*, *rpl16*, *petD*, *petB*, *atpF*, *rpoC1*, *clpP*) contained a single intron, while one gene, *ycf3*, contained two introns (**Table 2**). Additionally, four tRNA genes containing one intron were also identified as follows: *trnV^UAC^*, *trnA^UGC^*, *trnI^GAU^*, and *trnL^UAA^*. As observed in most legumes, the *infA*, *rpl22*, and *rps16* genes were lost (Lei et al., 2016). The overall GC content of the *V. sepium* chloroplast genome was 35.0%, whereas that of the protein-coding, intron, tRNA, rRNA and IGS regions was 36.7%, 34.6%, 52.3%, 54.2%, and 29.2%, respectively (**Table S4**). The RSCU result revealed that the *V. sepium* protein-coding sequences showed codon usage bias, with all preferred synonymous codons ending with A/T nucleotides and a high AT content at the 3^rd^ codon positions (72.2%) (**Figure S2**, **Table S4**).

**Table 1:** Genes predicted in the chloroplast genome of *V. sepium*.

Category	Group of genes	Names of genes
Self-replication	Large subunit of ribosomal proteins	*rpl2, rpl14, rpl16, rpl20, rpl23* ^a^, *rpl32, rpl33, rpl36*
	Small subunit of ribosomal proteins	*rps2, rps3, rps4, rps7, rps8, rps11, rps12* ^b^, *rps14, rps15, rps18, rps19*
	DNA dependent RNA polymerase	*rpoA, rpoB, rpoC1, rpoC2*
	rRNA genes	*rrn16S, rrn4.5S, rrn23S, rrn5S*
	tRNA genes	*trnA-TGC, trnC-GCA, trnD-GTC, trnE-TTC, trnF-GAA, trnG-TCC, trnH-GTG, trnI-CAT, trnI-GAT, trnK-TTT, trnL-CAA, trnL-TAA, trnL-TAG, trnM-CAT* ^c^, *trnMf-CAT, trnN-GTT, trnP-GGG, trnP-TGG, trnQ-TTG, trnR-ACG, trnR-TCT, trnS-GCT, trnS-GGA, trnS-TGA, trnT-GGT, trnV-TAC, trnW-CCA, trnY-GTA*
Photosynthesis	Photosystem I	*psaA, psaB, psaC, psaI, psaJ*
	Photosystem II	*psbA, psbB, psbC, psbD, psbE, psbF, psbH, psbI, psbJ, psbK, psbL, psbM, psbN, psbT, psbZ*
	NADH dehydrogenase	*ndhA, ndhB, ndhC, ndhD, ndhE, ndhF, ndhG, ndhH, ndhI, ndhJ, ndhK*
	Cytochrome b6/f complex	*petA, petB, petD, petG, petL, petN*
	ATP synthase	*atpA, atpB, atpE, atpF, atpH, atpI*
	Rubisco	*rbcL*
Other genes	Maturase	*matK*
	Protease	*clpP*
	Envelope membrane protein	*cemA*
	Subunit acetyl-CoA-carboxylase	*accD*
	C-type cytochrome synthesis gene	*ccsA*
Genes of unknow function	Conserved open reading frames	*ycf1, ycf2, ycf3, ycf4*

One open reading frame, *ORF292,* could not be annotated. ^a^pseudogene; ^b^trans-splicing gene; ^c^duplicated gene.

**Table 2:** Lengths of introns and exons of the split genes in the *V. sepium* complete chloroplast genome.

Gene name	Gene Location			Length (bp)				
	Strand	Start	End	Exon I	Intro I	Exon II	Intro II	Exon III
*ndhA*	-	17,922	20,213	552	1,200	540		
*ndhB*	+	39,164	41,349	720	674	792		
*rpl2*	+	49,205	50,732	393	700	435		
*rpl16*	+	52,173	53,655	9	1,072	402		
*petD*	-	57,360	58,556	9	714	474		
*petB*	-	58,753	60,207	6	804	645		
*atpF*	-	74,347	75,592	168	670	411		
*rpoC1*	-	83,263	86,132	435	791	1,644		
*clpP*	+	92,455	93,604	363	559	228		
*ycf3*	+	97,292	99,294	126	742	228	781	126
*trnV-UAC*	+	9,320	9,976	39	581	37		
*trnA-UGC*	-	32,593	33,473	38	808	35		
*trnI-GAU*	-	33,539	34,292	42	677	35		
*trnL-UAA*	+	119,177	119,535	37	272	50		

### SSRs and Repeats in *V. sepium*

**Figure 2:**
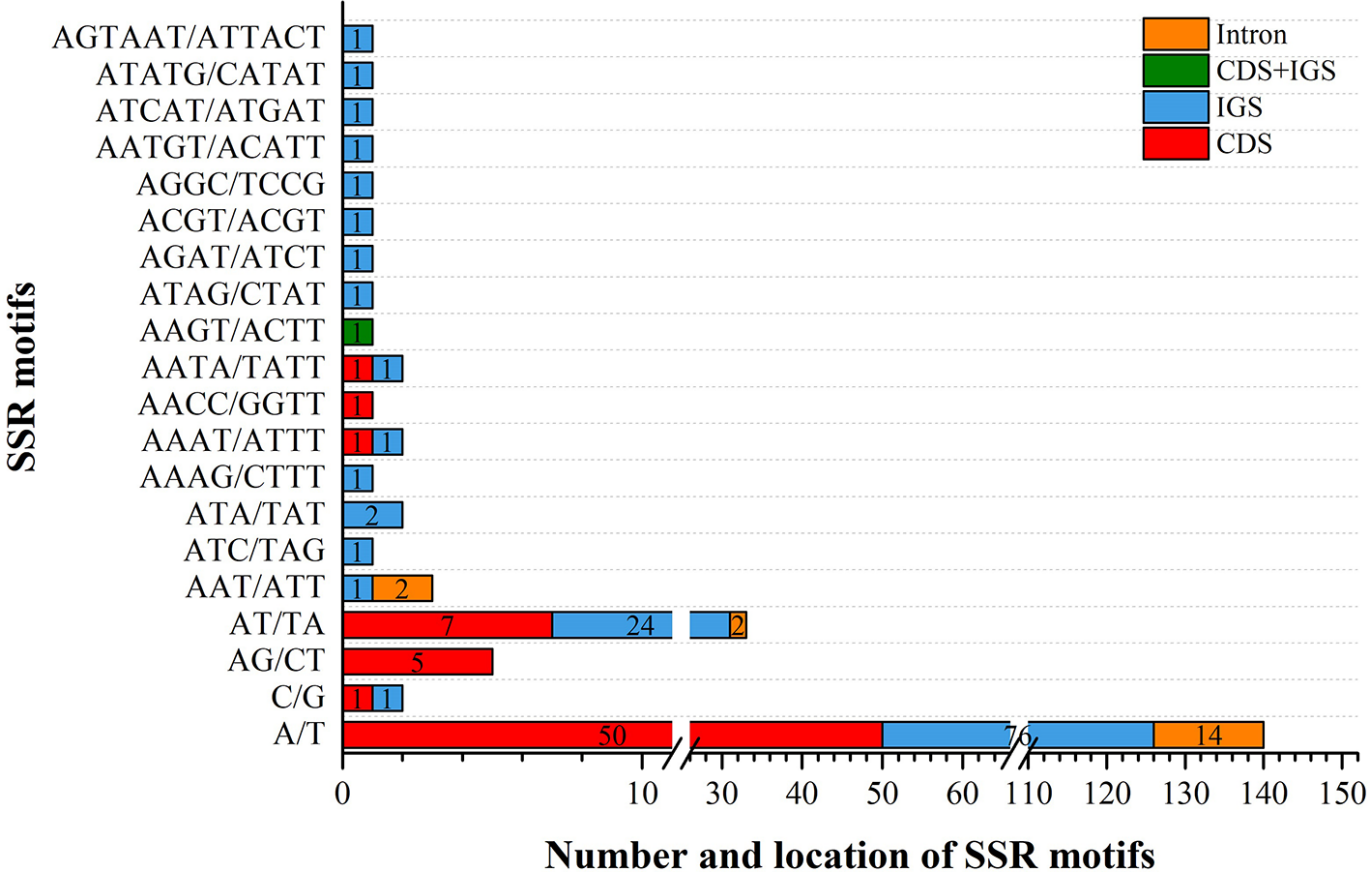
The types and distribution of SSRs along the chloroplast genome of *V. sepium*. Different locations, including CDS, IGS, CDS and IGS, and intron regions, are represented as colored boxes.

We analyzed the presence of SSRs and repeats in *V. sepium*. SSRs, which are regarded as useful gene markers, exhibited a high mutation rate. In this study, a total of 201 SSRs were found in the chloroplast genome of *V. sepium* (**Figure 2**). A majority of the SSRs were composed of mono-nucleotide and di-nucleotide repeat motifs. The types of SSRs distributed within the chloroplast genome of *V. sepium* were characterized, revealing that the SSR motifs of mono-nucleotide repeats mainly consisted of A/T (98.5%) and that those of di-nucleotide repeats mainly consisted of AT/TA (86.8%). A total of 116 and 66 V*. sepium* SSRs were distributed in the IGS and CDS regions, respectively (**Figure 2**).

Repeat sequences are essential for genome rearrangements, phylogenetic construction (Cavalier-Smith, 2002) and indel, and substitution variation (Yi et al., 2013). Sixty-two repeats, including 46 forward repeats, 4 palindromic repeats, and 12 tandem repeats, were found in the chloroplast genome of *V. sepium*. The lengths of the palindromic repeats were 45, 50, 54, and 155 bp, and the lengths of the forward repeats and tandem repeats ranged from 45 to 222 bp and 32 to 229 bp, respectively (**Table S5**). In addition, the maximum number of repeats (n = 49) were located in IGS regions, followed by those in CDSs (n = 27) (**Table S5**). We also found that most of these repeats were located in the *psaB*-*rps14* (n = 20), *ycf1*-*trnN-GUU* (n = 10), *accD* (n = 6) and *rps14* (n = 5) regions.

### Comparative Analyses of the Chloroplast Genomes of Fabeae Species

Twenty complete chloroplast genomes from within Fabeae were selected for comparison with *V. sepium.* One Cicereae species, *C. arietinum*, was set as the outgroup (**Table 3**). The changes in chloroplast genome length in these species ranged from 120, 289 bp (*L. odoratus*) to 126,421 bp (*L. pubescens*), and the greatest variation in length relative to *V. sepium* was 3.0% in the protein-coding region of *L. culinaris*, followed by the IGS region (2.8%) of *L. culinaris*. An average difference in length of only 0.1% was found in the tRNA and rRNA gene regions. Additionally, the GC content of the twenty-two complete chloroplast genomes ranged from 33.9% to 35.2%, exhibiting little change. After comparing *V. sepium* genes with those of twenty-one other Fabeae species, we found an inserted gene that is a unique unannotated protein-coding gene, *ORF292*, between *rps12* and *rps4* in *V. sepium*. Moreover, the *rps12* to *rps4* region in *V. sativa* also contained an inserted duplicated *rpl20* gene (not mentioned in the table). From genome rearrangement, we can infer that inversion events may result in gene insertion (**Figure S3**). We also found a pseudogene, *rpl23*, in *V. sepium*, *V. sativa*, *P. abyssinicum*, *P. sativum*, *P. sativum* subsp. Elatius and *L. sativus*. By analyzing gene and intron losses, all twenty-two species lost the *infA*, *rpl22*, and *rps16* genes, similar to most of the IR-lacking species. *Ycf4* genes were found in only *V. sepium*, *V. faba*, *P. sativum*, and *L. sativus*. Moreover, one intron of the *clpP* and *rpl16* genes was lost in *L. graminifolius* and *V. faba*, respectively (**Table 3**).

**Table 3:** Characteristics of twenty-one Fabeae species and *Cicer arietinum*.

Species	*V. sep*	*V. sat*	*V. fab*	*P. aby*	*P. sat*	*P. sat* ^sub^	*P. ful*	*L. cul*	*L. pub*	*L. ven*	*L. pal*	*L. jap*	*L. och*	*L. dav*	*L. lit*	*L. inc*	*L. gra*	*L. tin*	*L. cly*	*L. sat*	*L. odo*	*C. ari*
Length (bp)	12,4095	12,2467	123,722	12,2174	12,2169	12,1958	120,837	122,967	126,421	125,459	124,287	124,242	123,911	123,895	123,734	123,153	122,438	122,165	121,263	121,020	120,289	125,319
Genes	110	109	110	108	110	108	107	108	108	110	109	109	109	108	108	108	109	108	110	109	107	108
CDS genes^*^	77	76	76	75	76	75	73	74	74	75	75	75	75	74	74	74	75	74	75	75	73	75
tRNA genes	29	29	30	29	30	29	30	30	30	30	30	30	30	30	30	30	30	30	31	30	30	29
Introns**	14	14	14	14	15	14	15	14	15	15	15	15	15	14	15	15	14	14	15	15	15	15
CDS region	53.9%	52.9%	53.2%	53.9%	54.3%	53.9%	52.7%	50.9%	53.3%	52.2%	53.0%	52.9%	53.0%	52.6%	53.1%	54.0%	53.5%	53.5%	53.7%	55.3%	54.1%	52.3%
Intron region	8.9%	9.0%	8.8%	9.1%	9.7%	9.1%	9.2%	9.0%	8.8%	8.8%	8.8%	8.8%	8.8%	8.9%	8.9%	9.1%	8.5%	9.2%	9.3%	9.7%	9.2%	9.7%
IGS region	31.9%	32.7%	32.6%	31.5%	30.4%	31.5%	32.5%	34.7%	32.6%	33.7%	32.7%	32.8%	32.8%	33.1%	32.6%	31.4%	32.5%	31.8%	31.5%	29.4%	31.0%	32.6%
tRNA region	1.7%	1.7%	1.8%	1.8%	1.9%	1.8%	1.8%	1.7%	1.7%	1.8%	1.8%	1.8%	1.8%	1.8%	1.8%	1.8%	1.8%	1.8%	1.9%	1.9%	1.8%	1.8%
rRNA region	3.6%	3.7%	3.6%	3.7%	3.7%	3.7%	3.7%	3.7%	3.6%	3.6%	3.7%	3.7%	3.6%	3.7%	3.6%	3.7%	3.7%	3.7%	3.7%	3.8%	3.7%	3.6%
Genome GC	35.0%	35.2%	34.6%	34.8%	34.8%	34.8%	34.9%	34.4%	35.0%	34.9%	34.9%	34.9%	34.9%	34.9%	34.8%	34.7%	35.0%	34.9%	35.0%	35.1%	35.2%	33.9%
CDS GC	36.7%	36.9%	36.6%	36.8%	36.8%	36.8%	36.9%	36.6%	36.7%	36.7%	36.7%	36.7%	36.7%	36.8%	36.6%	36.6%	36.7%	36.7%	36.9%	36.9%	37.0%	36.3%
Intron GC	34.6%	34.7%	34.5%	34.4%	34.1%	34.4%	34.3%	34.1%	34.6%	34.7%	34.7%	34.7%	34.7%	34.7%	34.6%	34.1%	35.0%	34.3%	34.5%	34.2%	34.3%	33.5%
IGS GC	29.2%	29.4%	28.0%	28.2%	28.1%	28.2%	28.5%	28.4%	29.4%	29.1%	28.8%	28.9%	29.0%	28.9%	28.8%	28.4%	28.9%	28.8%	28.6%	28.6%	29.0%	27.0%
tRNA GC	52.3%	52.5%	52.4%	52.8%	52.7%	52.8%	52.7%	52.2%	52.4%	52.8%	52.7%	52.8%	52.8%	52.9%	52.8%	52.7%	52.8%	52.4%	52.6%	52.7%	52.6%	52.7%
rRNA GC	54.2%	54.2%	54.2%	54.0%	53.9%	54.0%	54.0%	53.8%	54.0%	54.2%	54.1%	54.1%	54.2%	53.9%	54.2%	54.1%	54.0%	54.0%	54.3%	53.6%	53.7%	54.2%
Gene losses		*ycf4*		*ycf4*		*ycf4*	*ycf4* *psaI* *accD*	*ycf4* *rps18* *psbZ*	*ycf4* *psaI*	*ycf4*	*ycf4*	*ycf4*	*ycf4*	*ycf4* *psaI*	*ycf4* *psbJ*	*ycf4* *psaI*	*ycf4*	*ycf4* *rps18*	*ycf4*	*psaI*	*ycf4* *rps18* *psaI*	*ycf4*
Gene gains	*ORF292*							*lhbA*														

All of these species contain four rRNA genes except for L. ven (L. venosus). The full names of the twenty-two species are as follows: V. sep, V. sepium; V. sat, V. sativa; V. fab, V. faba; P. aby, P. abyssinicum; P. sat, P. sativum; P. satsub, P. sativum subsp. Elatius; P. ful, P. fulvum; L. cul, L. culinaris; L. pub, L. pubescens; L. ven, L. venosus; L. pal, L. palustris; L. jap, L. japonicus; L. och, L. ochroleucus; L. dav, L. davidii; L. lit, L. littoralis; L. inc, L. inconspicuus; L. gra, L. graminifolius; L. tin, L. tingitanus; L. cly, L. clymenum; L. sat, L.sativus; L. odo, L.odoratus; C. ari, C. arietinum. *pseudogenes: rpl23 in V. sepium, V. sativa, P. abyssinicum, P. sativum, P. sativum subsp. Elatius and L. sativus; ycf1 in L. culinaris; ycf4 in P. sativum. **intron gains: one intron added to tRNA-Gly (V. faba, P. sativum, L. sativus, V. faba, C. arietinum) and ycf2 (P. fulvum, L. pubescens, L. venosus, L. palustris, L. japonicus, L. ochroleucus, L. littoralis, L. inconspicuus, L. graminifolius, L. clymenum, L. odoratus). intron losses: one intron missing in clpP (L. graminifolius) and rpl16 (V. faba).

**Figure 3:**
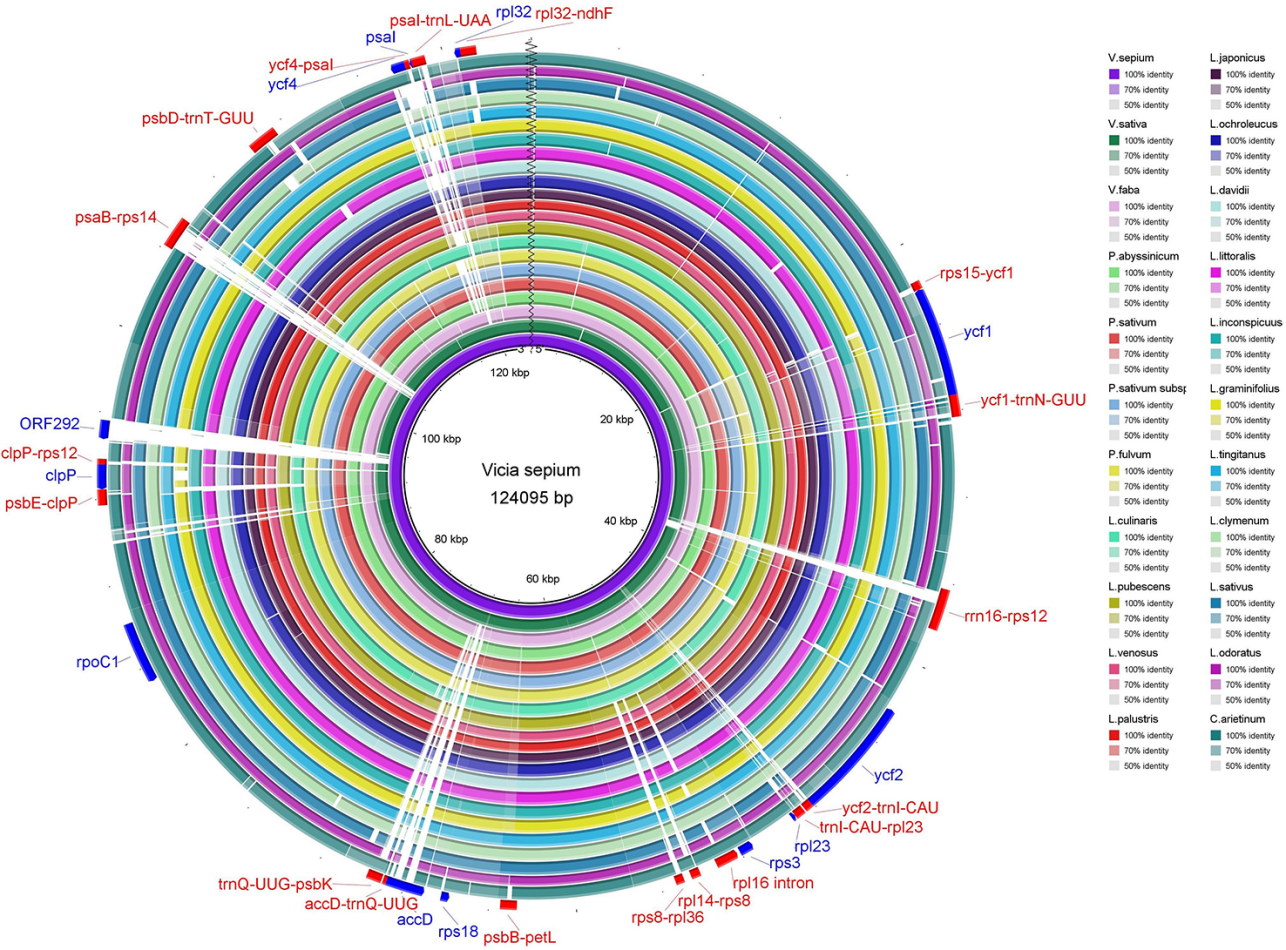
The sequence identity of 22 Fabaceae species. The inner circle is the reference genome. Next circles represent the sequence identity between *V. sepium* and 21 other species. The outermost circle corresponds to the protein-coding genes and intergenic spacer regions. Genes with clockwise arrows represent reverse strands, while genes with the counterclockwise arrow represent forward strands.

The sequence identity of the chloroplast genomes of *V. sepium* and twenty-one other Fabaceae species was visualized (**Figures 3** and **S4**), and the results revealed that coding regions are more highly conserved than noncoding regions. Usually, regions with 50% or less sequence identity can be regarded as highly divergent regions. In coding regions, *ycf1*, *ycf2*, *rpl23*, *rps3*, *rps18*, *accD*, *rpoC1*, *clpP*, *ORF292*, *ycf4, psaI*, and *rpl32* contained relatively low identity regions. In addition, these highly divergent noncoding regions include *rps15*-*ycf1*, *ycf1*-*trnN-GUU*, *rrn16*-*rps12*, *ycf2*-*trnI-CAU*, *trnI-CAU* -*rpl23*, *rpl16* intron, *rpl14*-*rps8*, *rps8*-*rpl36*, *psbB*-*petL*, *accD*-*trnQ-UUG*, *trnQ-UUG*-*psbK*, *psbE*-*clpP*, *clpP-rps12*, *psaB*-*rps14*, *psbD*-*trnT-GUU*, *ycf4*-*psaI*, *psaI*-*trnL-UAA*, and *rpl32-ndhF* (**Figure 3** and **S4**).

### Evolutionary Rate of Fabeae Species

The pairwise distances (K2P rates) of complete chloroplast genome sequences from twenty-one Fabeae species and one Cicereae species were calculated (**Table S6**). The results showed that the nucleotide variability rate ranged from 0.001 to 0.248 (*L. sativus* vs *C. arietinum*). Compared with *V. sepium*, the lowest K2P rate was 0.027 (*V. sativa*) while the highest K2P rate was found in *C. arietinum* (0.246) (**Table S6**). The mean K2P rate between *Pisum* and *V. sepium* was 0.217. The mean K2P rate between *Lathyrus* and *V. sepium* was 0.193. Specifically, the K2P rate between *V. faba* and *V. sepium* was 0.207, which was higher than the rate between *V. sepium* and some *Lathyrus* species. We hypothesized that *V. sepium* and *V. sativa* were located in the same clade and showed different evolutionary directions compared with *V. faba*.

**Figure 4:**
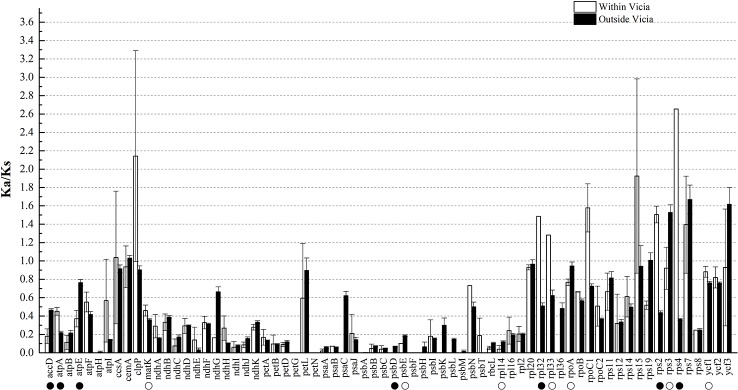
The Ka/Ks ratios of homologous protein-coding genes within and outside of the genus *Vicia* with *V. sepium* as the reference. White boxes represent the mean Ka/Ks values within the genus *Vicia*, and black boxes indicate the mean Ka/Ks values outside of the genus *Vicia*. The data are the arithmetic mean ± SE. Symbols under the gene names indicate levels of statistical significance between the species within *Vicia* and the species outside of *Vicia*: no symbol, *P* > 0.05, blank circle, *P* = 0.01–0.05; black circle, *P* < 0.01. The X-axis denotes the homologous genes.

Ka and Ks nucleotide substitutions within *Vicia* and outside of *Vicia* were calculated with *V. sepium* as the reference, as well as the Ka/Ks ratio (**Table S2**, **Figure 4**). The Ka/Ks ratio is an important parameter for determination of the selective constraint acting on each gene (Keller et al., 2017). Ka/Ks > 1 indicates that the gene was under positive selection, whereas Ka/Ks = 1 or <1 indicates genes under neutral selection or purifying selection (Kimura, 1980). The mean Ks between *V. sepium* and twenty Fabeae species ranged from 0.0058 (*petN*) to 0.2375 (*ycf1*), and the mean Ka ranged from 0 (*petG*, *psbF*) to 0.1846 (*clpP*) (**Table S2**). Within the genus *Vicia*, nine genes (*ccsA*, *clpP*, *rpl32*, *rpl33*, *rpoC1*, *rps15*, *rps2, rps4* and *rps7*) with a Ka/Ks ratio >1 (**Figure 4**) evolved under beneficial mutations, and 60 genes evolved under purifying selection, including sixteen genes that evolved almost neutrally, showing a ratio range of 0.5 to 1. Twelve conserved genes (*atpH*, *petG*, *petN*, *psaC*, *psbA*, *psbD*, *psbF*, *psbH*, *psbK*, *psbL*, *psbM* and *rpl36* with Ka/Ks = 0) presented a very strong purifying selective pressure. Comparison of sequence divergence between *Vicia* and other genera showed that the Ka/Ks ratios of the eight genes (*accD*, *atpA*, *matK*, *rpl32*, *rpl33*, *rps2*, *rps4*, *ycf1*) were significantly higher (*P* < 0.05) in *Vicia*, and among these genes, the ratios of *accD*, *atpA*, *rpl32*, *rps2* and *rps4* were extremely significantly higher (*P* < 0.01).

**Figure 5:**
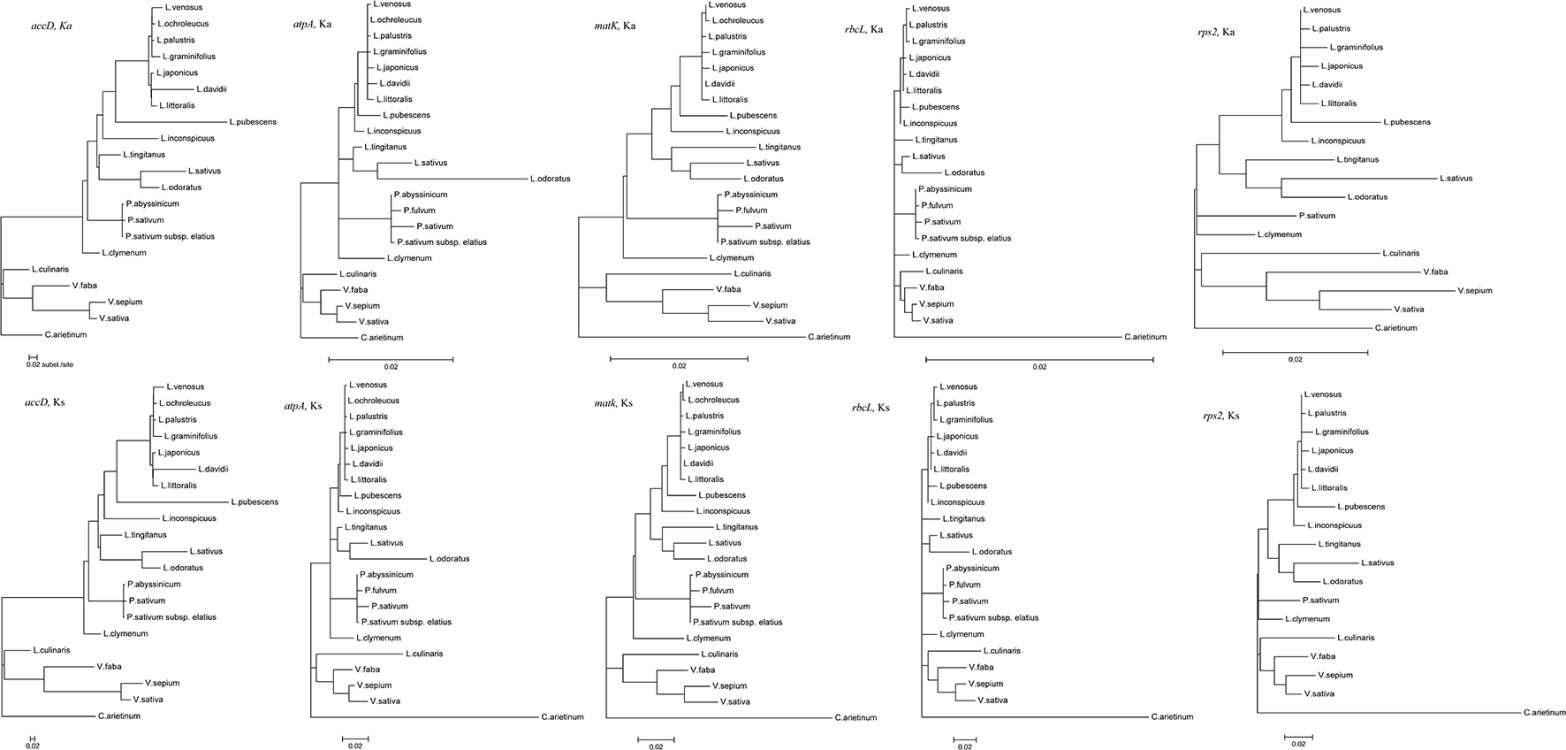
Synonymous and nonsynonymous divergence in the Fabeae chloroplast genes. All tree topologies were completely constrained as described in the Methods section. All trees were drawn to the same scale representing the number of substitutions per synonymous or nonsynonymous site.

Codon-based likelihood analysis (**Table S3**; **Figure S1**) was performed to compare the Ka/Ks ratios of the *accD*, *atpA*, *rpl32*, *rps2*, and *rps4* genes across different Fabeae lineages. *C. arietinum* was set as the reference. The null model (H0) hypothesized that the A0 (*Cicer*), A1 (*Pisum* and *Lathyrus*), A2 (*Lens* and *Vicia*), A3 (*Lens*), and A4 (*Vicia*) clades exhibit the same Ka/Ks ratio. The alternative model hypothesized that one or more clades present different Ka/Ks ratios. By comparing the *p*-values of the two different models, the results demonstrated that the best models for *accD*, *atpA*, *rpl32*, *rps2*, and *rps4* are H2, H3, H0, H2, and H0, respectively (**Table S3**). A higher Ka/Ks ratio in a specific clade is considered to indicate accelerated evolution of the clade. The Ka/Ks ratios of *accD*, *atpA* and *rps2* in the *Vicia* clade were higher than those in the *Cicer* clade, and the Ka/Ks ratios of *rpl32* and *rps4* were the same in the two clades. The results revealed that evolution rates increased in *atpA*, *rps2* and *accD* of the *Vicia* lineage but exhibited no change in *rpl32* or *rps4* (**Table S3**). The Ka/Ks ratios of *rps2* in the *Vicia* clade were higher than those in the *Pisum* and *Lathyrus* clade, but the *Vicia* clade presented lower Ka/Ks ratios in the *accD* and *atpA* genes. The results revealed that *rps2* exhibited a higher evolutionary rate in *Vicia*, while *atpA* and *accD* in *Pisum* and *Lathyrus* evolved much faster. We also compared the synonymous and nonsynonymous nucleotide substitution rates of genes that evolved rapidly (*accD*, *atpA*, and *rps2*) in different Fabeae lineages to the rates observed in genes that did not evolve rapidly (*rbcL* and *matK*) based on codon-based ML phylogenetic analysis. As shown in **Figure 5**, in the Ka and Ks trees, the substitutions per nonsynonymous site of *rps2* evolved much faster in *Vicia* than in other Fabeae species, but no similar acceleration was observed in *rbcL* and *matK*. In addition, all Fabeae lineages showed accelerated evolution in the *accD* gene for high synonymous and nonsynonymous nucleotide substitution rates compared to *rbcL* and *matK*. This result can supplement Magee’s findings (Magee et al., 2010). We also detected amino acid differences in the *accD*, *atpA*, *rps2*, *matK*, and *rbcL* genes within and outside of *Vicia* by aligning the sequences from Fabeae species (**Figures S5**–**S9**). Notably, there is less amino acid sequence conservation in *accD* (83.03% identity between *Vicia* species) and *rps2* (91.98% identity between *Vicia* species) than in *matK* (94.23% identity between *Vicia* species) and *rbcL* (99.16% identity between *Vicia* species). The lengths of the amino acid sequences ranged from 165 to 1,141 in *accD*.

### Phylogenetic Analysis of *V. sepium*

Considering the rather limited number of complete *Vicia* chloroplast genomes (only 3), it is difficult to determine whether *Vicia* is paraphyly. Therefore, in addition to the complete chloroplast genomes and conserved chloroplast protein-coding sequences, we constructed a phylogenetic tree of *Vicia* using two widely sequenced chloroplast genes, namely, *rbcL* and *matK*, to support our result. Detailed information regarding these four datasets can be found in **Table S1**. Upon comparing the four NJ trees, we found that *V. sepium*, *V. sativa*, and *V. faba* were located in the same evolutionary branch with support rates of 100% in the protein-coding sequence tree, 99% in the *matK* tree, and 49% in the *rbcL* tree. However, in the whole-genome tree, the result was different, with *V. sepium* and *V. sativa* located in the same clade and *V. faba* located in another clade. These results indicated that the evolutionary histories of *V. sepium* and *V. sativa* were similar but different from that of *V. faba* (**Figures S10**–**S12**). Both the *rbcL* and *matK* phylogenetic trees showed that *Vicia* species were included in different clades, which supports our hypothesis that *Vicia* is paraphyletic (**Figures S11** and **S12**).

**Figure 6:**
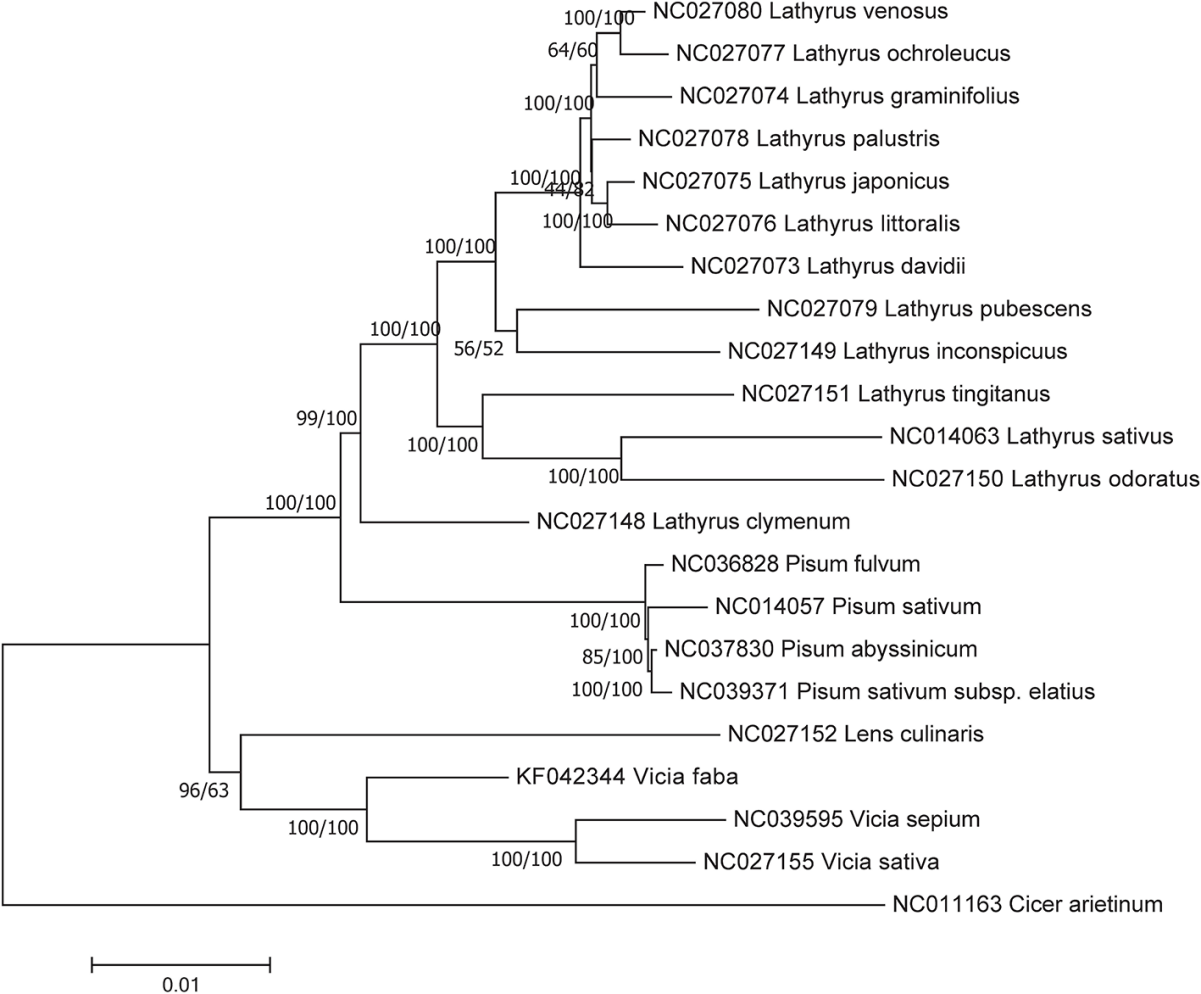
Phylogenetic relationships based on the conserved chloroplast protein-coding sequences of 21 Fabeae species and *C. arietinum* with the maximum likelihood (ML) method and the neighbor joining (NJ) method. *C. arietinum* was selected as the outgroup. Numbers on the left and right side at the branches represent bootstrap values of the ML method and the NJ method respectively.

Both the NJ and ML phylogenetic trees for homologous protein-coding sequences showed that *Vicia* and *Lens* were included in the same clade, together with *Pisum* and *Lathyrus* (**Figure 6**), but the ML tree presented a higher support rate for the *Vicia* and *Lens* clade than the NJ tree.

## Discussion

### Beneficial Gene Mutations Observed in the Protein-Coding Regions

In our study, within genus *Vicia*, *ccsA*, *clpP*, *rpl32*, *rpl33*, *rpoC1*, *rps15*, *rps2*, *rps4*, and *rps7* showed positive selection, with a Ka/Ks ratio >1 (**Figure 4**). None of these genes are related to photosynthesis (*psa*, *psb*, *ndh*, *pet*, *atp*). In fact, genes related to photosynthesis were under less selection pressure than other types of genes (Du et al., 2016; Li et al., 2017; Gao et al., 2018). Such positive selection is also found in other species, as observed for two genes flanking *ycf4* (*accD* and *cemA*) in *Lathyrus* (Magee et al., 2010); *accD*, *ycf1*, and *atpA* in seed plants (Zheng et al., 2017); *rps14* in *Dodonaea viscosa* and *Sapindus mukorossi* (Saina et al., 2018); and the *atpF* gene in two deciduous *Quercus* species (Yin et al., 2018). In general, genes under selection pressures are mainly identified by comparing the synonymous and nonsynonymous nucleotide substitution rates in related species. Thus, genes under positive selection pressure in different lineages can be identified. However, the positive selection acting on genes in a specific lineage contrasts with the silent molecular clock hypothesis, according to which the point mutation rate in all regions of the same genome is almost constant (Ochman and Wilson, 1987). The factors causing a higher Ka/Ks ratio in some sequences than in the rest of the genome remain unclear. Here, we consider two explanations for this difference. One possible explanation for this phenomenon is that a greater number of nucleotide substitutions are associated with gene duplications and gene losses. Erixon found that positive selection acting on the *clpP* gene in various plant lineages is related to repeated duplication (Erixon and Oxelman, 2008). Magee showed that the Ka/Ks ratios of *cemA* and *accD* flanking *ycf4* are >1 in *Lathyrus*. This may occur because the increase in the nucleotide mutation rate near the hypermutational *ycf4* gene affects the purifying selection acting on the amino acid sequence (Magee et al., 2010). Another possibility is that differential selection may act on gene divergence. For example, research on oak species showed that the *atpF* gene is highly divergent (Ka/Ks > 1) in the comparation between deciduous oak and evergreen sclerophyllous oak because the former loses its leaves in the cold and drought seasons (Yin et al., 2018). Another study on seed plants suggested that genes affected by positive selection are always involved in plant adaptation, such as *accD*, *ycf1* and *atpA* (Zheng et al., 2017).

We also found that *atpA*, *accD*, and *rps2* of *Vicia* showed significantly accelerated evolution (**Figures 4**, **5**, **S5**–**S7**, **Table S3**). *Rps2*, encoding the ribosomal protein S2, is retained in almost all plants. The exceptions mainly occur in Apocynaceae. For example, in milkweeds, a 2.4-kb mitochondrial DNA sequence was horizontally transferred to the *rps2*-*rpoC2* plastid intergenic region, resulting in two pseudogenes, namely, *rps2* and *rpoC2*, contained in plastomes (Straub et al., 2013). However, such plastome insertion is rare. A relatively common type of evolution is the point mutation described in our study. For example, Ka and Ks rates are elevated in parasitic Scrophulariaceae and Orobanchaceae, which provide suitable material for studying the evolution of hemi- and holoparasitic plant lineages (dePamphilis et al., 1997). In *Gossypium*, the Yrp8 and Cys11 sites of *rps2* and the other nine genes are undergoing protein sequence evolution, which may aid the adaptation of cotton species to diverse environments (Wu et al., 2018). The accelerated evolution of *atpA* (participating in ATP synthesis) has also been found in other species, such as Dipsacales (Fan et al., 2018) and *Urophysa* (Xie et al., 2018) species. Consistent with our study, only one to three sites show positive selection. *AccD* is essential for plant leaf development and has been lost in some angiosperm lineages. It is believed that *accD* was functionally transferred to the nucleus (Magee et al., 2010; Sabir et al., 2014).

At present, *Vicia* is the only known legume genus in which so many genes show positive selection and accelerated evolution in the chloroplast genome. Therefore, a comprehensive understanding of the mechanism underlying the increased nucleotide substitution of homologous protein-coding genes is necessary, and *Vicia* species may be suitable model systems for such studies.

### Genome Variation in the Chloroplast Genomes of *V. sepium*

To detect the genome variation in the chloroplast genome of *V. sepium*, we compared *V. sepium* with related genera in the tribe Fabeae. Our results revealed that the greatest variation in genome length relative to *V. sepium* was located in protein-coding regions (**Table 3**). This finding is consistent with Zheng’s research (Zheng et al., 2017), showing that chloroplast gene length is an important factor affecting chloroplast genome size based on phylogenetic signals. The length variation of protein-coding regions may result from gene loss and gain or differences in the lengths of homologous genes. *Ycf4*, encoding a photosystem I assembly protein, is the most easily deleted gene in Fabeae species (**Table 3**). This result supports previous findings revealing that *ycf4* has been lost in many species of *Lathyrus* and *Pisum* due to its functional transfer to the nuclear genome (Magee et al., 2010). Furthermore, gene insertion events involving one new unannotated protein-coding gene, namely, *ORF292* (879 bp) and one duplicated gene, namely, *rpl20* (354 bp), were found in *V. sepium and V. sativa*, respectively. One pseudogene, *rpl23*, was identified in *V. sepium* and *V. sativa* (**Table 3**). This indicates that the evolutionary histories of *V. sepium* and *V. sativa* are similar and that *V. faba* may be located in a different evolutionary clade. In general, a chloroplast gene cannot be lost arbitrarily unless the function of the gene is transferred to the nuclear genome or replaced by that of a nuclear gene (Magee et al., 2010). Therefore, the mechanism of loss of the *rpl23* gene in *V. sepium* and *V. sativa* requires further in-depth research. In addition to gene loss, one intron was also missing in *clpP* (*L. graminifolius*) and *rpl16* (*V. faba*) (**Table 3**). The *clpP* gene normally contains two introns in angiosperms (Jansen et al., 2007; Jansen et al., 2008). Jansen determined that the IRLC lineage (in which Fabeae is included) has lost one intron of *clpP* (Jansen et al., 2008). However, the loss of two introns observed in *clpP* is rare; Sabir’s research (Sabir et al., 2014) on the IRLC lineage (in which Fabeae is included) showed that this phenomenon has only occurred in *Glycyrrhiza glabra*, and our findings are complementary to this previous work. *V. faba* was the only species found to have lost the intron of *rpl16* in the tribe Fabeae, and the *rpl16* intron shows high divergence in *Chusquea* (Kelchner and Clark, 1997), *Gleditsia* (Schnabel and Wendel, 1998), and Cacteae (Butterworth et al., 2002). This result indicates that different evolutionary clades exist in *Vicia*. In addition to gene loss and gain, differences in the lengths of homologous genes are also found in Fabeae species (ranging from 495 to 3,423, 36 to 537, and 3,879 to 5,403 in *accD*, *rps12* and *ycf1*, respectively). In seed plants, the length difference in *atpA*, *accD*, and *ycf1* is the main reason for chloroplast genome size variation (Zheng et al., 2017).

In addition to protein-coding region expansion and contraction in *V. sepium*, protein-coding sequence divergence also exists. In our study, the GC content of the chloroplast genome of *V. sepium* was found to be lower than that of other species, such as *Chikusichloa mutica* [tribe rice (Wu et al., 2017)], *Arabidopsis thaliana* [Brassicaceae (Asaf et al., 2017a)], and *Quercus aquifolioides* [Fagaceae (Yin et al., 2018)], which exhibit a conserved structure and evolution of the chloroplast genome (**Table S4**). Normally, a higher GC content indicates a more stable genome sequence (Wu et al., 2017). Therefore, to consider the genome variation in *V. sepium* protein-coding regions, we surveyed SSRs, repeat loci, highly divergent regions and pairwise sequence divergence. Many SSRs and repeat loci appeared in the protein-coding regions (CDSs) (**Table S5**, **Figure 2**). These results are consistent with previous reports on *Astragalus membranaceus* (Lei et al., 2016). Because of the slippage of DNA strands, SSRs, regarded as useful gene markers, present a high mutation rate (Huang et al., 2018). Repeated sequences are believed to result in aberrant replication and repair pathways (Sabir et al., 2014). The genes *ycf1*, *ycf2*, *rpl23*, rps3, *rpl18*, *accD*, *rpoC1*, *clpP*, *ORF292*, *ycf4, psaI*, and *rpl32* share relatively low identity (**Figures 3** and **S4**). *V. sepium* showed considerable differences from other Fabeae species (with the exception of *V. sativa*), even *V. faba*. Therefore, *Vicia* presents profound genome variation, which is significant for the evolutionary history of the chloroplast genome.

### Evolution in *Vicia*

The phylogenetic analysis conducted with the conserved chloroplast protein-coding sequences of *rbcL* and *matK* showed that *Vicia* and *Lens* were included in the same clade (**Figures 6** and **S12**). This result is also supported by the synapomorphy that is observable in the currently available research. *Vicia* and *Lens* both produce the phytoalexin wyerone, which is not found in *Pisum* and *Lathyrus* (Schaefer et al., 2012), and show high average protein richness and *in vitro* protein digestibility (Pastor-Cavada et al., 2014). However, even within *Vicia*, different evolutionary directions can be found, resulting in the paraphyly of *Vicia*. For example, in our study, the pairwise distance between *V. sepium* and *V. sativa* was much greater than that between *V. sepium* and *V. faba* (**Table S6**). The former species also showed a gene insertion in the *rps12* to *rps4* region (**Figure S3**) and an accelerated evolutionary rate in *accD* (**Figure 5**). In addition to chloroplast genome characteristics, the life form, stylar characteristics, and chromosome numbers of these species support this result. Ancestral *Vicia* species originating from the Mediterranean shared an annual life form, a basic chromosome number of 2n=14 and evenly hairy styles. However, the recent evolutionary reconstruction of *Vicia* indicates that a perennial life form, a chromosome number of 2n=12 (or 10, 24, 28, 42) and adaxially/abaxially hairy styles have arisen in *Vicia* (Schaefer et al., 2012). In the comparison of *Vicia* species in our study, all of the species were found to produce adaxially hairy styles, but *V. sepium* has evolved a perennial life form, while *V. sativa* and *V. faba* share the same characteristic of an annual life form. Nevertheless, the evolution of the life form of *Vicia* verified that *V. sepium* and *V. sativa* had a shared evolutionary history. Therefore, we can infer from all of these results that *Vicia* species may adopt different evolutionary strategies and that the chloroplast genome provides ideal material for reconstructing the evolutionary history of *Vicia*.

In summary, a new chloroplast genomic resource for an important wild resource plant, *V. sepium*, is presented. This study fills the gap in *V. sepium* genomic resources and provides novel insights into evolutionary dynamics in a poorly studied *Vicia* clade. Our results reveal that *Vicia* species may have experienced many instances of positive selection in the chloroplast genome and accelerated evolution of protein-coding genes, which is rare, being found in only a few angiosperm species. Detailed surveys show that *V. sepium* presents profound genomic variation in terms of *ORF292* gene insertion, *rpl23* pseudogene detection, lower GC content, CDS length variation, and accelerated evolution of the *atpA*, *accD*, and *rps2* genes. Analysis of the phylogenetic relationships show that *Vicia* and *Lens* are included in the same clade and that the evolutionary direction of *V. sepium* and *V. sativa* is different from that of *V. faba*. Therefore, *Vicia* species may be a suitable model system for understanding the mechanisms of chloroplast genome evolution. This study is expected to attract researchers toward *Vicia* species, leading to the identification of further evidence regarding the evolutionary history of the chloroplast genome.

## Data Availability Statement

All datasets generated for this study are included in the article/**Supplementary Material**.

## Author Contributions

CL, ZX, and GY conceived the study. All authors collected field samples. CL, JP, and XP analyzed the final data. YZ acquired funds (2016NK2148, 2016TP2007) for this study. CL wrote the original manuscript, and all authors commented on an early draft of the manuscript.

## Funding

This work was supported by the Major Science and Technology Program of Hunan Province (2017NK1014), Key Technology R&D Program of Hunan Province (2016NK2148, 2016TP2007, 2017TP2006), Forestry Science and Technology Project of Hunan Province (XLK201825, XLK201920) and Natural Science Foundation of Hunan Province (2019JJ50027).

## Conflict of Interest

The authors declare that the research was conducted in the absence of any commercial or financial relationships that could be construed as a potential conflict of interest.

## Supplementary Material

Figure S1 Topology of Fabeae lineages obtained from a concatenated data set consisting of *matK* and *rbcL. C. arietinum* was selected as the out group.

Figure S2 Codon usage and relative synonymous codon usage (RSCU) of the *V. sepium* chloroplast genome. The color of the histogram corresponds to the color of the codon. The size of the histogram corresponds to the RSCU of the codon. The X-axis represents different amino acids and the associated codons.

Figure S3 Genomic rearrangement of six Fabeae species relative to *V. sepium*. Locally collinear blocks (LCBs) are colored to indicate syntenic regions. Blocks below the center line indicate regions that align in the reverse complement (inverse) orientation. The small boxes below the LCBs of each chloroplast genome are represented as genes.

Figure S4 Alignment visualization of twenty-two Fabaceae complete chloroplast genomes using *V. sepium* as a reference. The vertical scale indicates the percent identity, ranging from 50% to 100%. Arrows indicate the annotated genes and their transcriptional direction. The different colored boxes correspond to exons, tRNA or rRNA, and noncoding sequences (CNSs).

Figure S5 Alignments of the *accD* protein sequences from Fabeae species.

Figure S6 Alignments of the *atpA* protein sequences from Fabeae species.

Figure S7 Alignments of the *rps2* protein sequences from Fabeae species.

Figure S8 Alignments of the *matK* protein sequences from Fabeae species.

Figure S9 Alignments of the *rbcL* protein sequences from Fabeae species.

Figure S10 Phylogenetic relationships based on the complete chloroplast genomes of twenty-two related species obtained by the neighbor joining (NJ) method. C. arietinum was selected as the outgroup.

Figure S11 Phylogenetic relationships based on *rbcL* gene sequences of 50 Fabeae species, *T. pretense* and *T. repens* obtained by the neighbor joining (NJ) method. *T. pretense* and *T. repens* were selected as the outgroup.

Figure S12 Phylogenetic relationships based on *matK* gene sequences of 62 Fabeae species, *T. pretense* and *T. repens* obtained by the neighbor joining (NJ) method. *T. pretense* and *T. repens* were selected as the outgroup.
