# Supplementary figures and images for: Initial Characterization of the Chloroplast Genome of Vicia sepium, an Important Wild Resource Plant, and Related Inferences About Its Evolution
Source: Front Genet. 2020 Feb 20;11:73. doi: 10.3389/fgene.2020.00073 (PMC7044246; doi:10.3389/fgene.2020.00073)

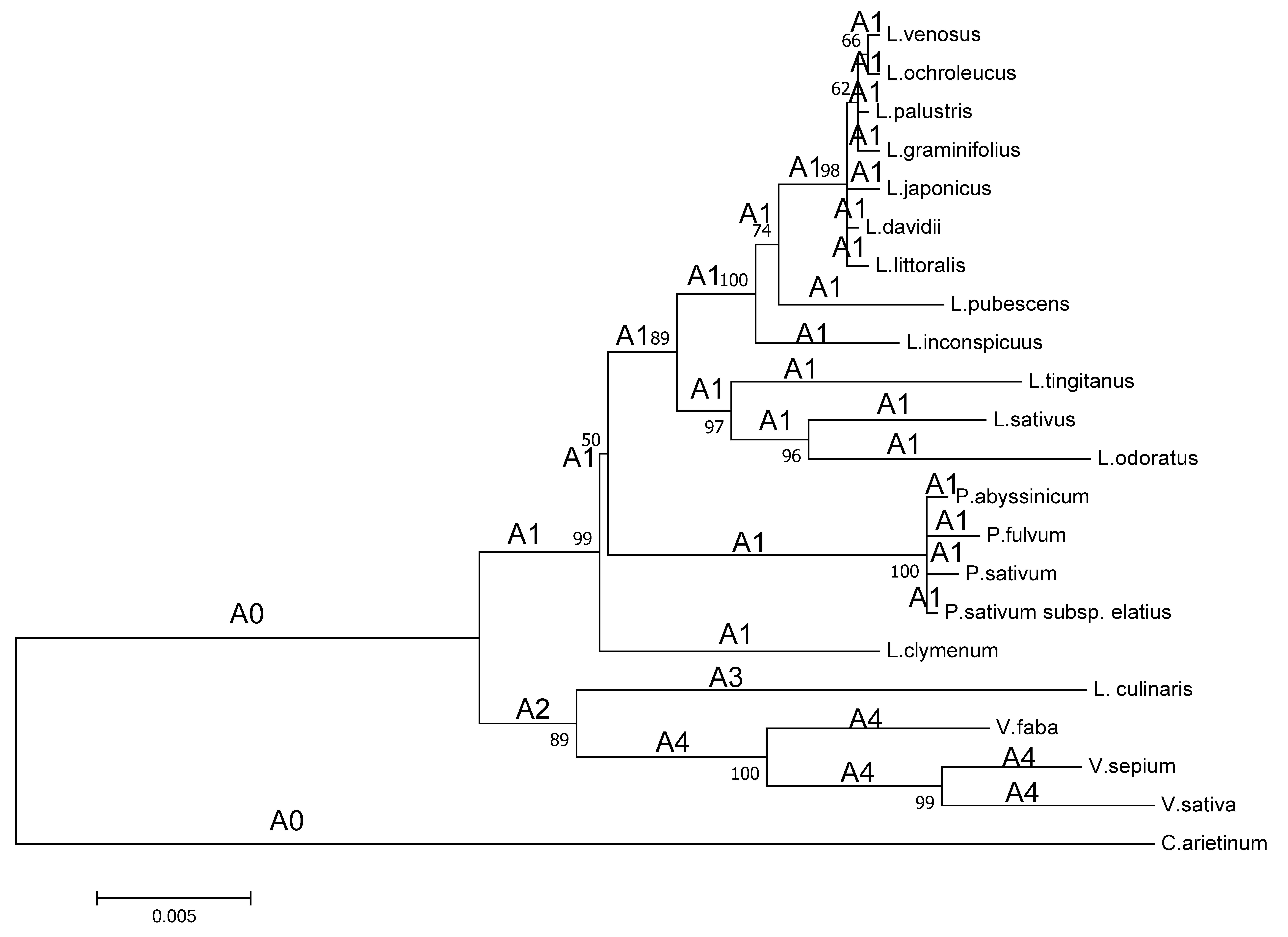

Supplement: Figure S1 — Topology of Fabeae lineages obtained from a concatenated data set consisting of matK and rbcL. C. arietinum was selected as the out group. [file Image_1.jpeg]

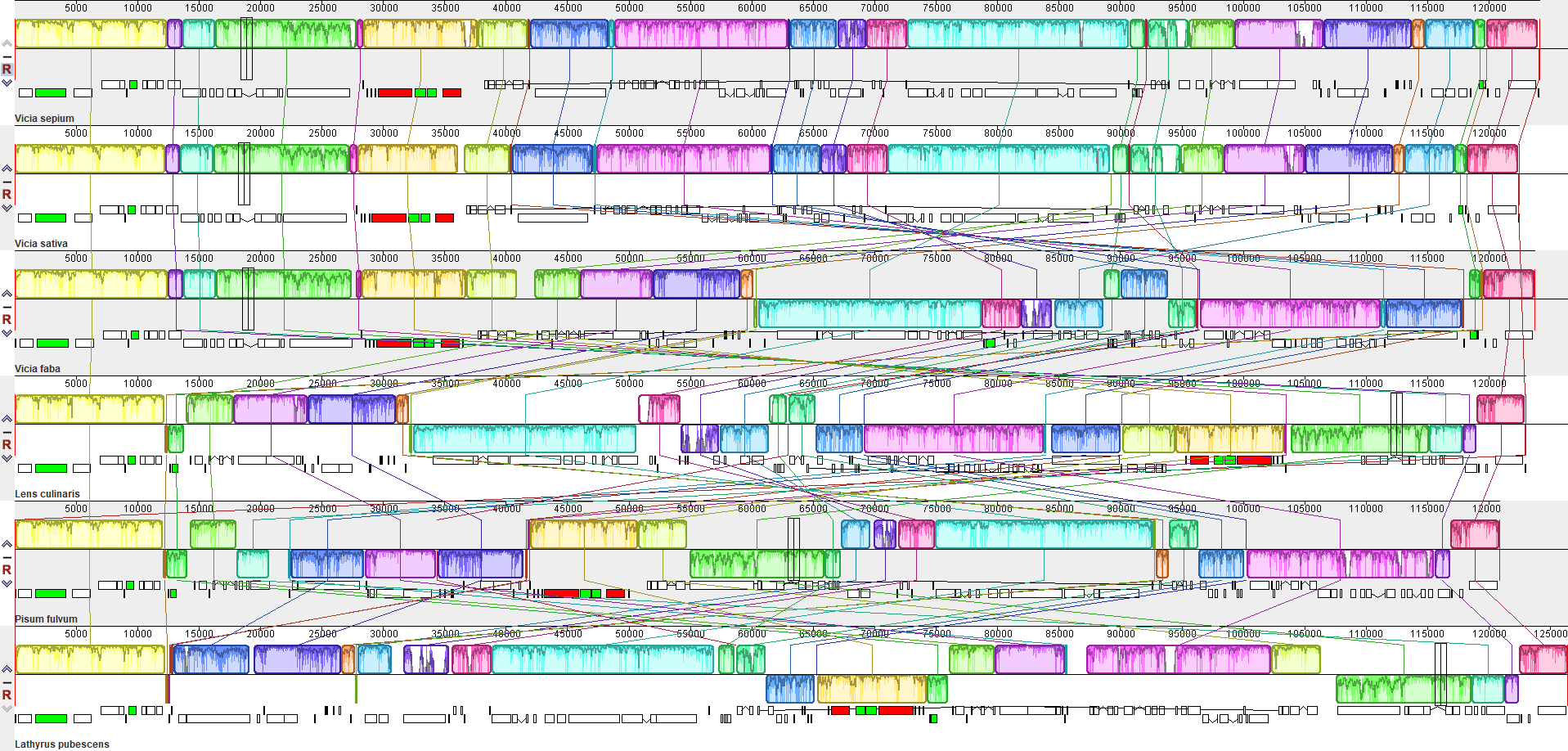

Supplement: Figure S3 — Genomic rearrangement of six Fabeae species relative to V. sepium. Locally collinear blocks (LCBs) are colored to indicate syntenic regions. Blocks below the center line indicate regions that align in the reverse complement (inverse) orientation. The small boxes below the LCBs of each chloroplast genome are represented as genes. [file Image_3.png]

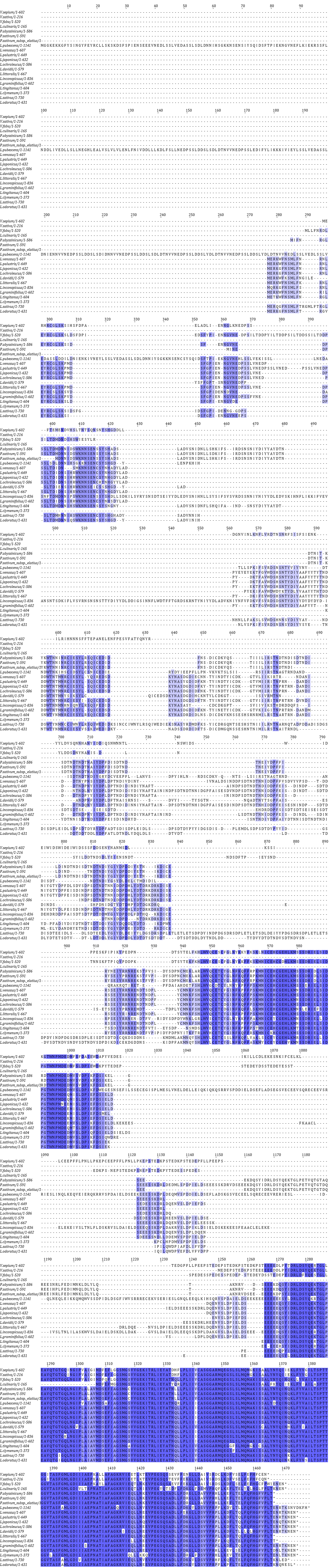

Supplement: Figure S5 — Alignments of the accD protein sequences from Fabeae species. [file Image_5.png]

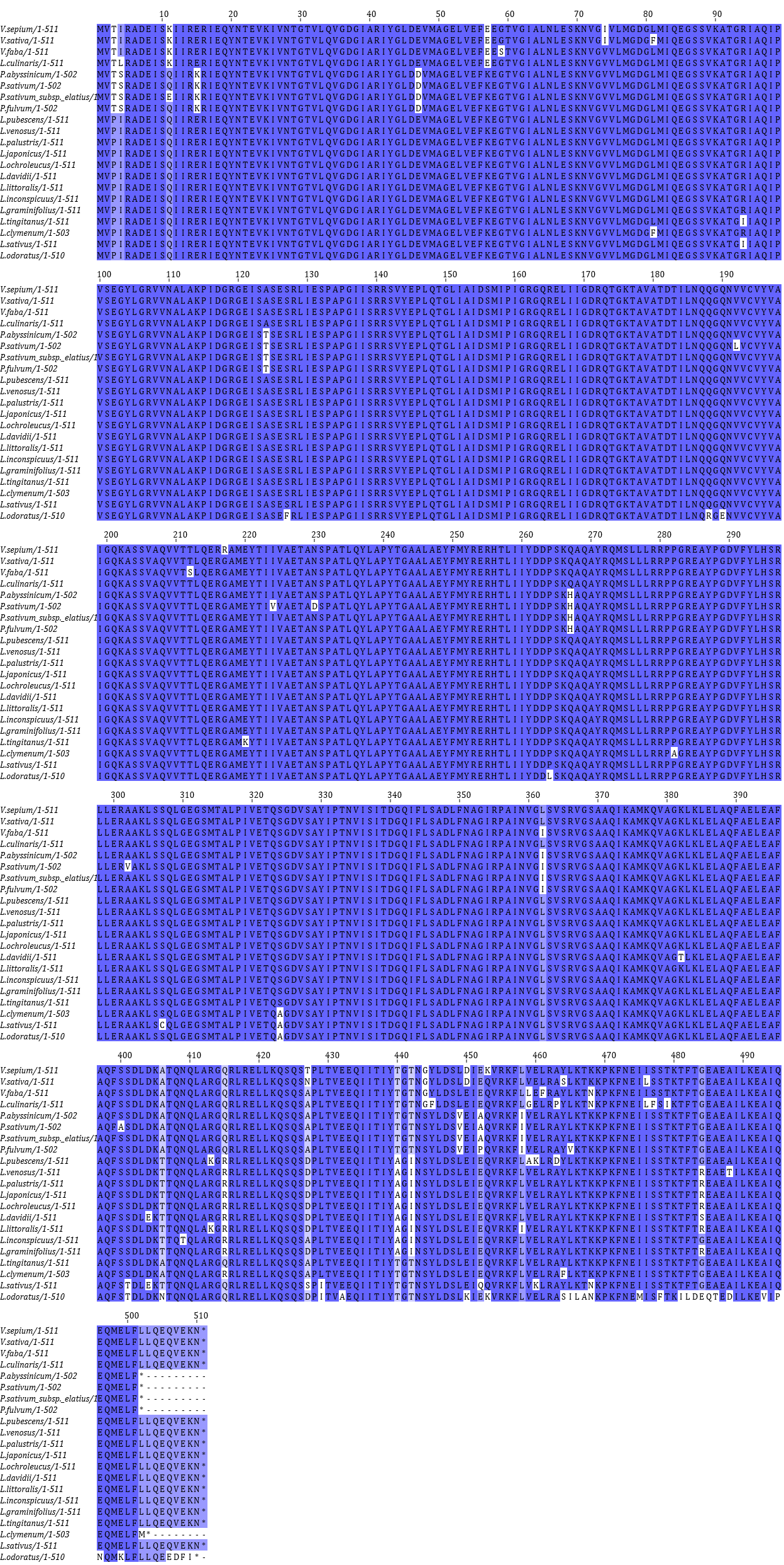

Supplement: Figure S6 — Alignments of the atpA protein sequences from Fabeae species. [file Image_6.png]

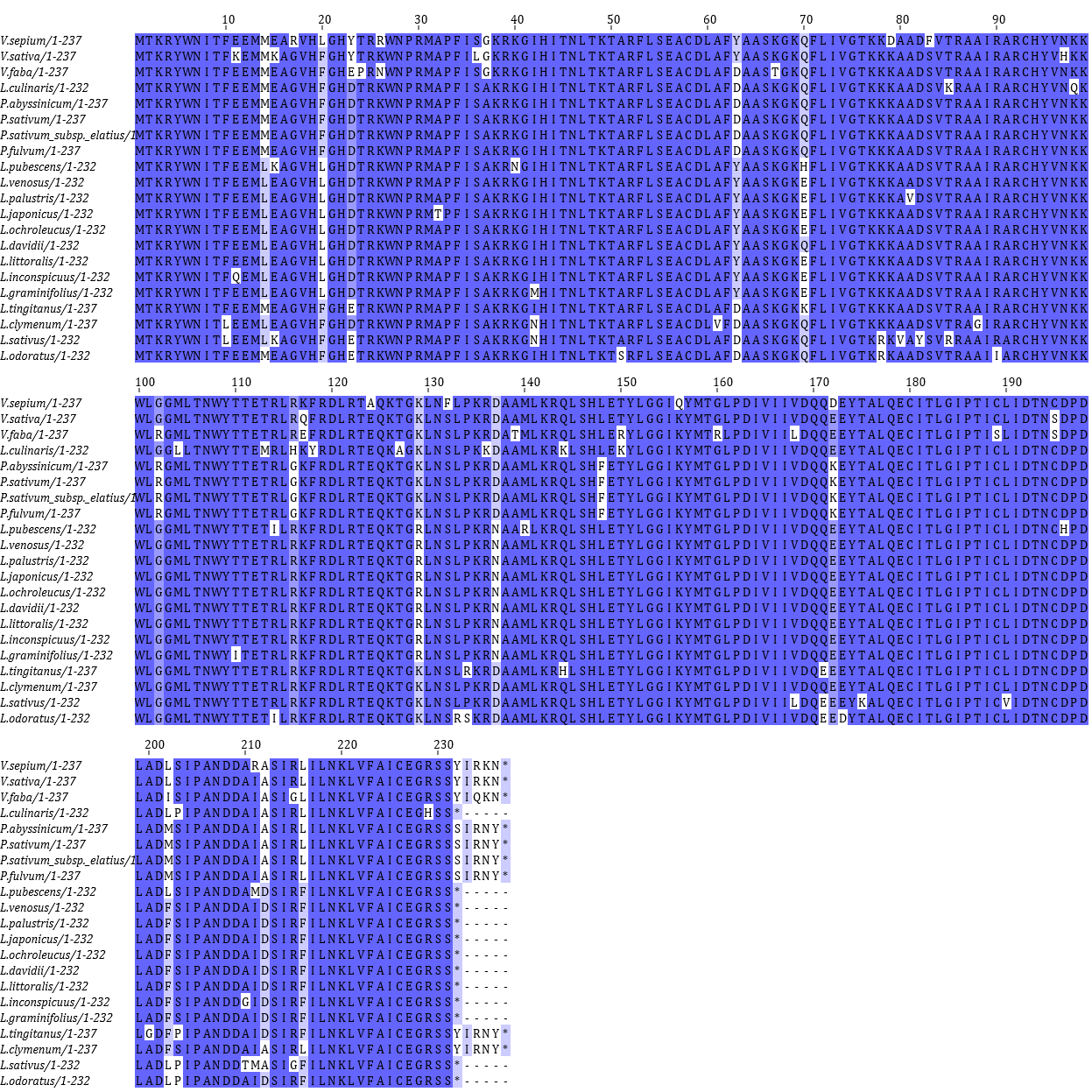

Supplement: Figure S7 — Alignments of the rps2 protein sequences from Fabeae species. [file Image_7.png]

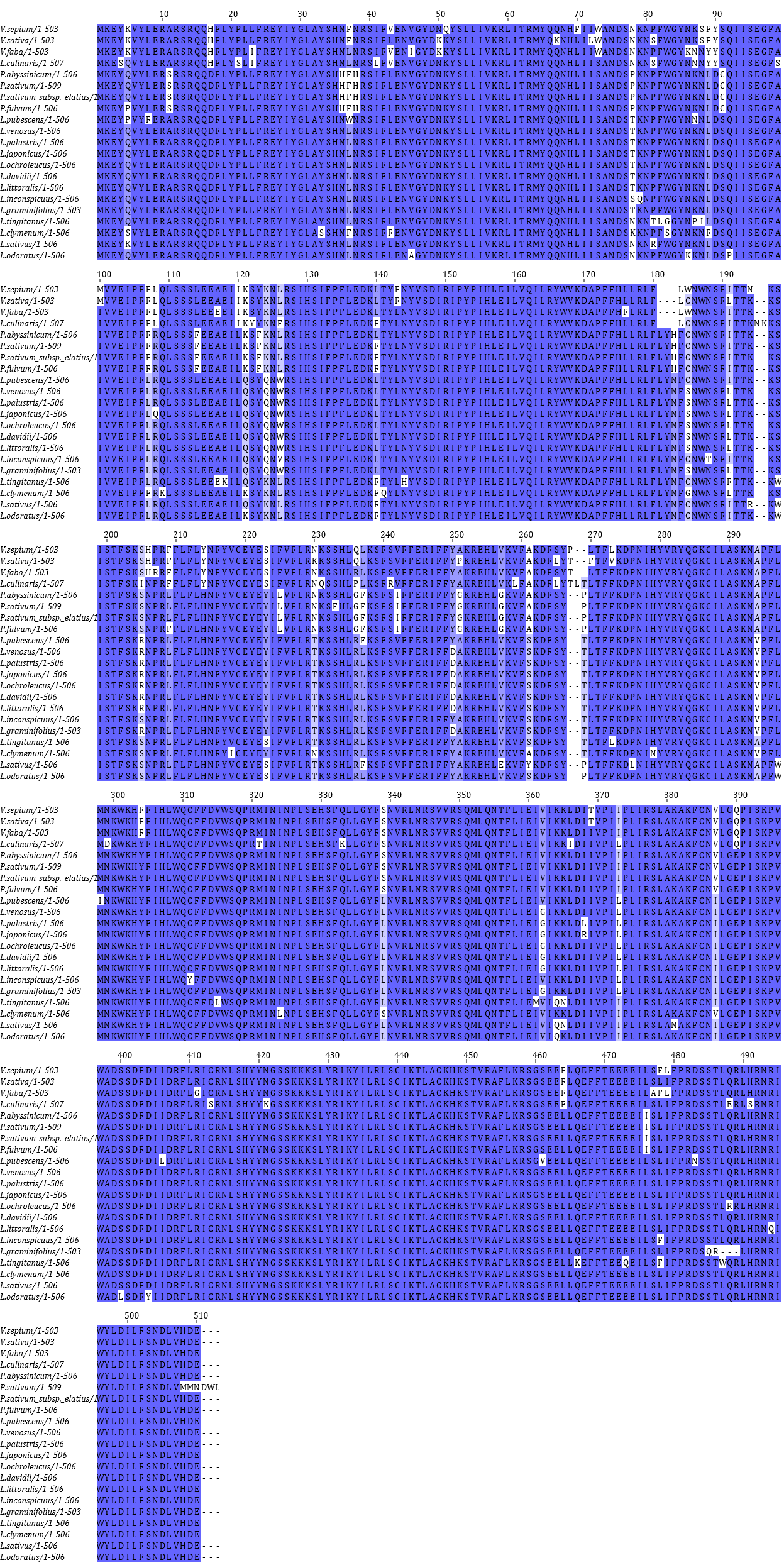

Supplement: Figure S8 — Alignments of the matK protein sequences from Fabeae species. [file Image_8.png]

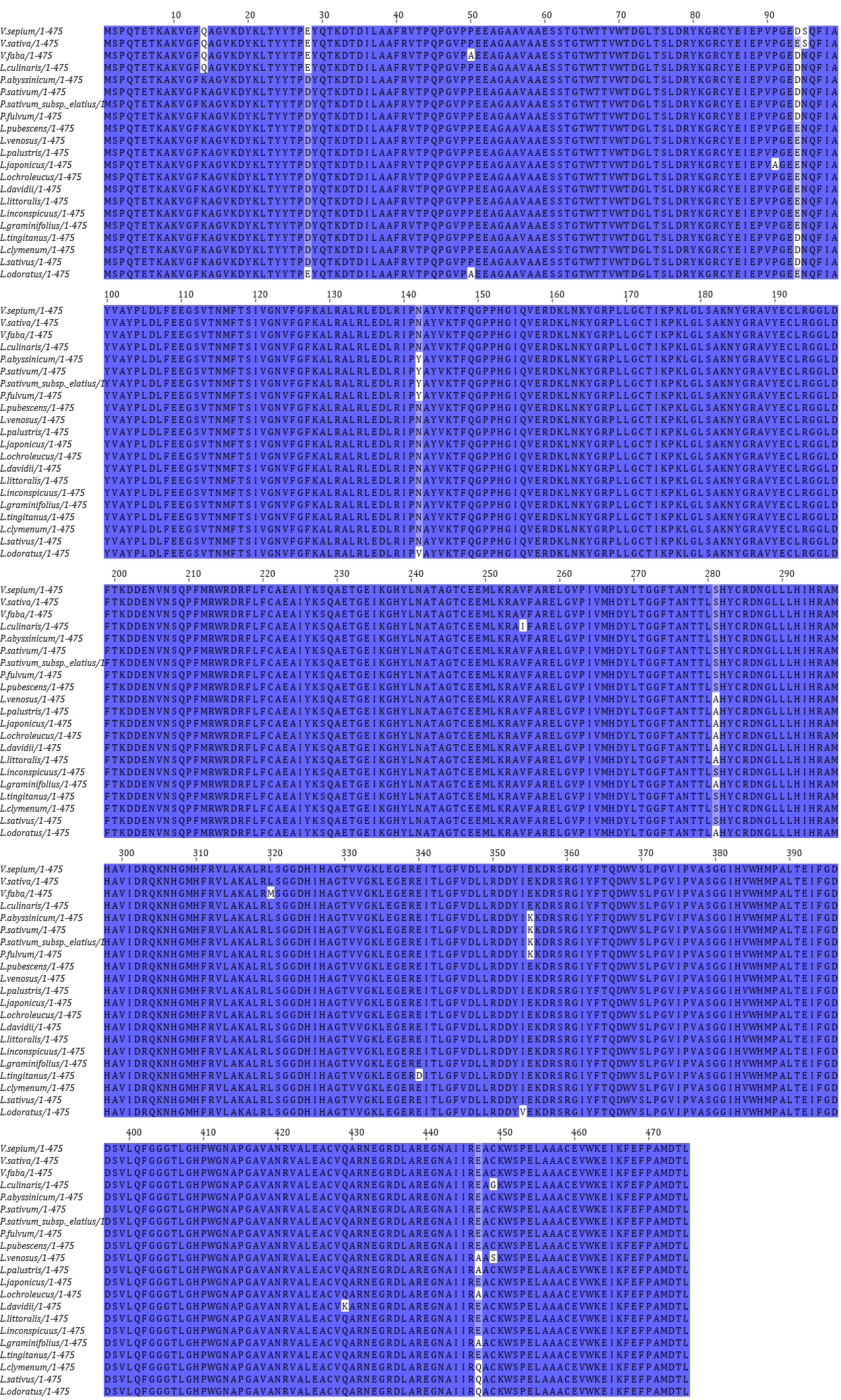

Supplement: Figure S9 — Alignments of the rbcL protein sequences from Fabeae species. [file Image_9.png]

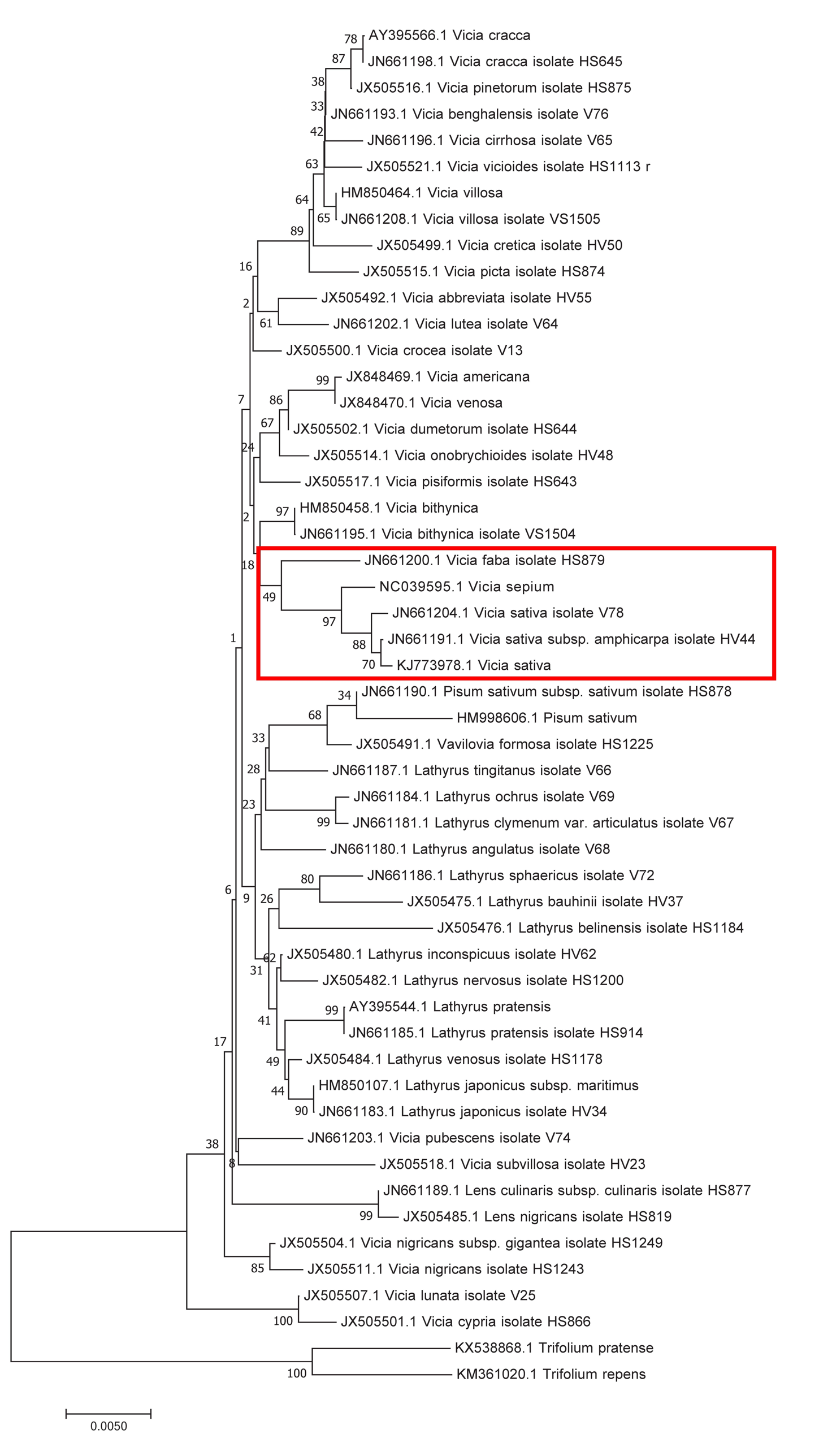

Supplement: Figure S11 — Phylogenetic relationships based on rbcL gene sequences of 50 Fabeae species, T. pretense and T. repens obtained by the neighbor joining (NJ) method. T. pretense and T. repens were selected as the outgroup. [file Image_11.jpeg]
