# Supplementary material for: Initial Characterization of the Chloroplast Genome of Vicia sepium, an Important Wild Resource Plant, and Related Inferences About Its Evolution
Source: Front Genet. 2020 Feb 20;11:73. doi: 10.3389/fgene.2020.00073 (PMC7044246; doi:10.3389/fgene.2020.00073)
Supplement: Supplementary file 15 [file Table_3.docx]

**Table S3**. Parameter estimates under a one-ratio model (H0) or a two-ratio model (H1-H5).

*accD*:

| Model^a^ | A0 | A1 | A2 | A3 | A4 | lnL | *P* |
| --- | --- | --- | --- | --- | --- | --- | --- |
| H0: A0=A1=A2=A3=A4 | 0.5109 | =A0 | =A0 | =A0 | =A0 | -19394.02 | NA |
| H1: A0=A1＝A2=A3≠A4 | 0.5351 | =A0 | =A0 | =A0 | 0.4075 | -19391.90 | *P*=0.04^b^ |
| H2: A0≠A1＝A2=A3≠A4 | 0.2479 | 0.5613 | =A1 | =A1 | 0.4161 | -19385.83 | *P*=0.0003^c^ |
| H3: A0≠A1≠A2=A3≠A4 | 0.2429 | 0.5554 | 1.2758 | =A2 | 0.4111 | -19384.64 | *P*=0.12^d^ |
| H4: A0≠A1=A2≠A3≠A4 | 0.2462 | 0.5564 | NA | 1.1484 | 0.4160 | -19384.93 | *P*=0.18^e^ |

^a^The topology and branch specific Ka/Ks ratios are presented in Figure S1.

^b^H0 v H1: df = 1

^c^H0 v H2: df = 2

^d^H2 v H3: df = 1

^e^H2 v H4: df = 1

*atpA*:

| Model^a^ | A0 | A1 | A2 | A3 | A4 | lnL | *P* |
| --- | --- | --- | --- | --- | --- | --- | --- |
| H0: A0=A1=A2=A3=A4 | 0.2148 | =A0 | =A0 | =A0 | =A0 | -3746.03 | NA |
| H1: A0=A1＝A2=A3≠A4 | 0.2147 | =A0 | =A0 | =A0 | 0.2157 | -3746.01 | *P*=0.84^b^ |
| H2: A0≠A1＝A2=A3≠A4 | 0.0518 | 0.3194 | =A1 | =A1 | 0.2124 | -3731.53 | *P*=0.00001^c^ |
| H3: A0≠A1≠A2=A3≠A4 | 0.0521 | 0.4056 | 0.0813 | =A2 | 0.2153 | -3725.09 | *P*=0.0003^d^ |
| H4: A0≠A1≠A2≠A3≠A4 | 0.0522 | 0.4066 | 0.0001 | 0.0884 | 0.2163 | -3724.56 | *P*=0.30^e^ |

^a^The topology and branch specific Ka/Ks ratios are presented in Figure S1.

^b^H0 v H1: df = 1

^c^H0 v H2: df = 2

^d^H2 v H3: df = 1

^e^H3 v H4: df = 1

*rpl32*:

| Model^a^ | A0 | A1 | A2 | A3 | A4 | lnL | *P* |
| --- | --- | --- | --- | --- | --- | --- | --- |
| H0: A0=A1=A2=A3=A4 | 0.5338 | =A0 | =A0 | =A0 | =A0 | -1131.00 | NA |
| H1: A0=A1＝A2=A3≠A4 | 0.3698 | =A0 | =A0 | =A0 | 0.7421 | -1129.84 | *P*=0.13^b^ |
| H2: A0≠A1＝A2=A3≠A4 | 0.1949 | 0.4314 | =A1 | =A1 | 0.7312 | -1129.35 | *P*=0.19^c^ |
| H3: A0=A1≠A2=A3=A4 | 0.3468 | =A0 | 0.7659 | =A2 | =A2 | -1129.50 | *P*=0.08^d^ |
| H4: A0=A1=A2≠A3=A4 | 0.3698 | =A0 | =A0 | 0.7421 | =A3 | -1129.84 | *P*=0.13^e^ |
| H5: A0≠A1=A2=A3=A4 | 0.1919 | 0.5853 | =A1 | =A1 | =A1 | -1129.92 | *P*=0.14^f^ |

^a^The topology and branch specific Ka/Ks ratios are presented in Figure S1. *P. abyssinicum* = *P. sativum* subsp. elatius; *L. venosus* = *L. palustris* = *L. japonicus* = *L. ochroleucus* = *L. davidii* = *L. littoralis* = L. inconspicuous = *L. graminifoliusm* = *L. tingitanus*

^b^H0 v H1: df = 1

^c^H0 v H2: df = 2

^d^H0 v H3: df = 1

^e^H0 v H4: df = 1

^f^H0 v H5: df = 1

*rps2*:

| Model^a^ | A0 | A1 | A2 | A3 | A4 | lnL | *P* |
| --- | --- | --- | --- | --- | --- | --- | --- |
| H0: A0=A1=A2=A3=A4 | 0.4168 | =A0 | =A0 | =A0 | =A0 | -2129.10 | NA |
| H1: A0=A1＝A2=A3≠A4 | 0.3442 | =A0 | =A0 | =A0 | 0.8739 | -2126.38 | *P*=0.02^b^ |
| H2: A0≠A1＝A2=A3≠A4 | 0.1305 | 0.4650 | =A1 | =A1 | 0.8916 | -2121.58 | *P*=0.0005^c^ |
| H3: A0≠A1≠A2=A3≠A4 | 0.1293 | 0.5039 | 0.3233 | =A2 | 0.9025 | -2121.27 | *P*=0.43^d^ |
| H4: A0≠A1=A2≠A3≠A4 | 0.1299 | 0.5050 | =A1 | 0.3206 | 0.9023 | -2121.23 | *P*=0.40^e^ |

^a^The topology and branch specific Ka/Ks ratios are presented in Figure S1. *P. abyssinicum* = *P. sativum* = *P. sativum* subsp. Elatius = *P. fulvum*; L. venosus = *L. ochroleucus*

^b^H0 v H1: df = 1

^c^H0 v H2: df = 2

^d^H2 v H3: df = 1

^e^H2 v H4: df = 1

*rps4*:

| Model^a^ | A0 | A1 | A2 | A3 | A4 | lnL | *P* |
| --- | --- | --- | --- | --- | --- | --- | --- |
| H0: A0=A1=A2=A3=A4 | 0.5817 | =A0 | =A0 | =A0 | =A0 | -1937.74 | NA |
| H1: A0=A1＝A2=A3≠A4 | 0.5127 | =A0 | =A0 | =A0 | 1.0552 | -1936.54 | *P*=0.12^b^ |
| H2: A0≠A1＝A2=A3≠A4 | 0.3561 | 0.5621 | =A1 | =A1 | 1.0550 | -1936.08 | *P*=0.19^c^ |
| H3: A0=A1≠A2=A3=A4 | 0.5415 | =A0 | 0.7509 | =A2 | =A2 | -1937.43 | *P*=0.43^d^ |
| H4: A0=A1=A2≠A3=A4 | 0.5415 | =A0 | =A0 | 0.7509 | =A3 | -1937.43 | *P*=0.43^e^ |
| H5: A0≠A1=A2=A3=A4 | 0.3550 | 0.6417 | =A1 | =A1 | =A1 | -1936.93 | *P*=0.20^f^ |

^a^The topology and branch specific Ka/Ks ratios are presented in Figure S1. *P. sativum* = *P. sativum* subsp. elatius; *L. venosus* = *L. palustris*

^b^H0 v H1: df = 1

^c^H0 v H2: df = 2

^d^H0 v H3: df = 1

^e^H0 v H4: df = 1

^f^H0 v H5: df = 1

*matK*:

| Model^a^ | A0 | A1 | A2 | A3 | A4 | lnL | *P* |
| --- | --- | --- | --- | --- | --- | --- | --- |
| H0: A0=A1=A2=A3=A4 | 0.3942 | =A0 | =A0 | =A0 | =A0 | -4307.53 | NA |
| H1: A0=A1＝A2=A3≠A4 | 0.3933 | =A0 | =A0 | =A0 | 0.3981 | -4307.53 | *P*=1.0^b^ |
| H2: A0≠A1＝A2=A3≠A4 | 0.2638 | 0.4462 | =A1 | =A1 | 0.3981 | -4306.02 | *P*=0.22^c^ |
| H3: A0=A1≠A2=A3=A4 | 0.3786 | =A0 | 0.4350 | =A2 | =A2 | -4307.39 | *P*=0.60^d^ |
| H4: A0=A1=A2≠A3=A4 | 0.3783 | =A0 | =A0 | 0.4403 | =A3 | -4307.36 | *P*=0.56^e^ |
| H5: A0≠A1=A2=A3=A4 | 0.2638 | 0.4346 | =A1 | =A1 | =A1 | -4306.09 | *P*=0.09^f^ |

^a^The topology and branch specific Ka/Ks ratios are presented in Figure S1.

^b^H0 v H1: df = 1

^c^H0 v H2: df = 2

^d^H0 v H3: df = 1

^e^H0 v H4: df = 1

^f^H0 v H5: df = 1

*rbcL*:

| Model^a^ | A0 | A1 | A2 | A3 | A4 | lnL | *P* |
| --- | --- | --- | --- | --- | --- | --- | --- |
| H0: A0=A1=A2=A3=A4 | 0.0837 | =A0 | =A0 | =A0 | =A0 | -2821.54 | NA |
| H1: A0=A1＝A2=A3≠A4 | 0.0912 | =A0 | =A0 | =A0 | 0.0464 | -2820.79 | *P*=0.22^b^ |
| H2: A0≠A1＝A2=A3≠A4 | 0.0761 | 0.1082 | =A1 | =A1 | 0.0464 | -2820.36 | *P*=0.31^c^ |
| H3: A0=A1≠A2=A3=A4 | 0.0937 | =A0 | 0.0563 | =A2 | =A2 | -2827.59 | *P*=1^d^ |
| H4: A0=A1=A2≠A3=A4 | 0.0992 | =A0 | =A0 | 0.0424 | =A3 | -2819.84 | *P*=0.07^e^ |
| H5: A0≠A1=A2=A3=A4 | 0.0762 | 0.0894 | =A1 | =A1 | =A1 | -2828.19 | *P*=1^f^ |

^a^The topology and branch specific Ka/Ks ratios are presented in Figure S1.

^b^H0 v H1: df = 1

^c^H0 v H2: df = 2

^d^H0 v H3: df = 1

^e^H0 v H4: df = 1

^f^H0 v H5: df = 1
