# Supplementary material for: Initial Characterization of the Chloroplast Genome of Vicia sepium, an Important Wild Resource Plant, and Related Inferences About Its Evolution
Source: Front Genet. 2020 Feb 20;11:73. doi: 10.3389/fgene.2020.00073 (PMC7044246; doi:10.3389/fgene.2020.00073)
Supplement: Supplementary file 16 [file Table_4.docx]

**Table S4.** Base compositions in the *V. sepium* chloroplast genome.

| Location | T/U (%) | C (%) | A (%) | G (%) | GC (%) | Length (bp) |
| --- | --- | --- | --- | --- | --- | --- |
| Genome | 32.5 | 18.1 | 32.5 | 16.9 | 35.0 | 124095 |
| tRNA genes | 23.1 | 26.5 | 24.7 | 25.8 | 52.3 | 2061 |
| rRNA gens | 26.5 | 31.1 | 19.3 | 23.1 | 54.2 | 4499 |
| introns region | 31.3 | 17.2 | 34.2 | 17.3 | 34.6 | 11045 |
| Protein-coding genes | 31.4 | 19.0 | 31.8 | 17.8 | 36.7 | 66913 |
| Intergenic spacer | 35.9 | 15.0 | 35.0 | 14.2 | 29.2 | 39577 |
| 1st positon | 24.2 | 18.1 | 30.7 | 27.1 | 45.2 | 22261 |
| 2nd positon | 33.5 | 20.1 | 29.3 | 17.1 | 37.2 | 22261 |
| 1st+2nd positon | 28.8 | 19.1 | 30.0 | 22.1 | 41.2 | 44522 |
| 3rd positon | 39.3 | 12.7 | 32.9 | 15.1 | 27.9 | 22261 |
