# Supplementary material for: Initial Characterization of the Chloroplast Genome of Vicia sepium, an Important Wild Resource Plant, and Related Inferences About Its Evolution
Source: Front Genet. 2020 Feb 20;11:73. doi: 10.3389/fgene.2020.00073 (PMC7044246; doi:10.3389/fgene.2020.00073)
Supplement: Supplementary file 17 [file Table_5.docx]

**Table S5.** Repeat sequences in the chloroplast genome of *V. sepium*.

| Number | Repeat type | Length (bp) | Location |
| --- | --- | --- | --- |
| 1 | Forward | 45 | IGS (*psaB*-*rps14*) |
| 2 | Forward | 46 | IGS (*ycf1*-*trnN-GUU*; *rpoB-trnC*-*GCA*) |
| 3 | Forward | 47 | IGS (*ycf1*-*trnN-GUU*; *rpoB-trnC*-*GCA*) |
| 4 | Forward | 47 | CDS (*accD*) |
| 5 | Forward | 52 | IGS (*rrn16-rps12*) |
| 6 | Forward | 52 | IGS (*trnQ-UUG; trnQ-UUG-psbK*) |
| 7 | Forward | 53 | IGS (*psaB*-*rps14*) |
| 8 | Forward | 53 | CDS (*accD*) |
| 9 | Forward | 53 | IGS (*psaB*-*rps14*) |
| 10 | Forward | 53 | IGS (*psaB*-*rps14*) |
| 11 | Forward | 54 | CDS (*rps18*) |
| 12 | Forward | 55 | IGS (*ycf1-trnN-GUU*); CDS (*rpoC1*) |
| 13 | Forward | 55 | IGS (*ycf1-trnN-GUU*; *rpoC1*-*rpoB*) |
| 14 | Forward | 55 | IGS (*psaB*-*rps14*) |
| 15 | Forward | 56 | IGS (*psaB*-*rps14*) |
| 16 | Forward | 57 | CDS (*rps18*) |
| 17 | Forward | 57 | CDS (*accD*) |
| 18 | Forward | 57 | IGS (*psaB*-*rps14*) |
| 19 | Forward | 59 | IGS (*ycf4-psaI; psaI-trnL-UAA*) |
| 20 | Forward | 59 | IGS (*psaB*-*rps14*) |
| 21 | Forward | 60 | IGS (*psaB*-*rps14*) |
| 22 | Forward | 63 | CDS (*accD*) |
| 23 | Forward | 64 | IGS (*ycf1-trnN-GUU*); CDS (*rpoC1*) |
| 24 | Forward | 65 | CDS (*psaA*; *psaB*) |
| 25 | Forward | 68 | IGS (*psaB*-*rps14*) |
| 26 | Forward | 71 | CDS (*ycf2*); IGS (*trnI-CAU-rpl23*) |
| 27 | Forward | 72 | IGS (*ycf1-trnN-GUU*; *rpoB-trnC-GCA*) |
| 28 | Forward | 74 | CDS (*accD*) |
| 29 | Forward | 80 | IGS (*psaB*-*rps14*) |
| 30 | Forward | 81 | IGS (*ycf1-trnN-GUU*; *rpoB-trnC-GCA*) |
| 31 | Forward | 82 | IGS (*rrn16-rps12*) |
| 32 | Forward | 82 | IGS (*psaB*-*rps14*); CDS (*rps14*) |
| 33 | Forward | 85 | IGS (*psaB*-*rps14*) |
| 34 | Forward | 90 | IGS (*rpoB-trnC-GCA*) |
| 35 | Forward | 90 | IGS (*psaB*-*rps14*); CDS (*rps14*) |
| 36 | Forward | 91 | IGS (*ycf1-trnN-GUU*; *rpoB-trnC-GCA*) |
| 37 | Forward | 95 | IGS (*psaB*-*rps14*) |
| 38 | Forward | 102 | IGS (*ycf2-trnI-CAU*) |
| 39 | Forward | 106 | CDS (*ycf2*); IGS (*trnI-CAU-rpl23*) |
| 40 | Forward | 108 | IGS (*ycf1-trnN-GUU*); CDS (*rpoB*) |
| 41 | Forward | 108 | CDS (*ycf2*); IGS (*trnI-CAU-rpl23*) |
| 42 | Forward | 110 | CDS (*psaB*-*rps14*) |
| 43 | Forward | 123 | IGS (*rrn16-rps12*) |
| 44 | Forward | 129 | IGS (*psaB*-*rps14*); CDS (*rps14*) |
| 45 | Forward | 137 | IGS (*psaB*-*rps14*); CDS (*rps14*) |
| 46 | Forward | 222 | IGS (*rpoC1*-*rpoB;* *rpoB-trnC-GCA*); CDS (*rpoB*) |
| 47 | Palindrome | 45 | IGS (*rpl20*-*accD*; *rps12*-*ORF292*) |
| 48 | Palindrome | 50 | Intron (*ndhA*, *rpl16*) |
| 49 | Palindrome | 54 | IGS (*rpl20*-*accD*; *rps12*-*ORF292*) |
| 50 | Palindrome | 155 | IGS (*ycf1*-*trnN-GUU*); Intron (*ndhB*) |
| 51 | Tandem | 32 | CDS (*ycf4*); IGS (*cemA*-*ycf4*) |
| 52 | Tandem | 32 | IGS (*trnI-GAU*-*rrn16*) |
| 53 | Tandem | 42 | CDS (*ycf2*) |
| 54 | Tandem | 45 | CDS (*accD*) |
| 55 | Tandem | 47 | CDS (*rps14*); IGS (*psaB*-*rps14*) |
| 56 | Tandem | 58 | IGS (*psaB*-*rps14*) |
| 57 | Tandem | 60 | CDS (*rps18*) |
| 58 | Tandem | 79 | CDS (*trnQ-UUG*); IGS (*trnQ-UUG*-*psbK*) |
| 59 | Tandem | 85 | IGS (*rpoB*-*trnC-GCA*) |
| 60 | Tandem | 110 | IGS (*ycf2*-*trnI-CAU*) |
| 61 | Tandem | 157 | IGS (*rrn16*-*rps12*) |
| 62 | Tandem | 229 | CDS (*rpoB*); IGS (*rpoC1-rpoB*) |
