# Supplementary material for: Initial Characterization of the Chloroplast Genome of Vicia sepium, an Important Wild Resource Plant, and Related Inferences About Its Evolution
Source: Front Genet. 2020 Feb 20;11:73. doi: 10.3389/fgene.2020.00073 (PMC7044246; doi:10.3389/fgene.2020.00073)
Supplement: Supplementary file 18 [file Table_6.docx]

**Table S6.** Average pairwise sequence distances of Fabeae species.

| Species | *V. sep* | *V. sat* | *V. fab* | *P. aby* | *P. sat* | *P. sat*^sub^ | *P. ful* | *L. cul* | *L. pub* | *L. ven* | *L. pal* | *L. jap* | *L. och* | *L. dav* | *L. lit* | *L. inc* | *L. gra* | *L. tin* | *L. cly* | *L. sat* | *L. odo* | *C. ari* |
| --- | --- | --- | --- | --- | --- | --- | --- | --- | --- | --- | --- | --- | --- | --- | --- | --- | --- | --- | --- | --- | --- | --- |
| *V. sep* |  |  |  |  |  |  |  |  |  |  |  |  |  |  |  |  |  |  |  |  |  |  |
| *V. sat* | 0.027 |  |  |  |  |  |  |  |  |  |  |  |  |  |  |  |  |  |  |  |  |  |
| *V. fab* | 0.207 | 0.208 |  |  |  |  |  |  |  |  |  |  |  |  |  |  |  |  |  |  |  |  |
| *P. aby* | 0.216 | 0.216 | 0.222 |  |  |  |  |  |  |  |  |  |  |  |  |  |  |  |  |  |  |  |
| *P. sat* | 0.217 | 0.216 | 0.222 | 0.002 |  |  |  |  |  |  |  |  |  |  |  |  |  |  |  |  |  |  |
| *P. sat*^sub^ | 0.216 | 0.216 | 0.222 | 0.001 | 0.001 |  |  |  |  |  |  |  |  |  |  |  |  |  |  |  |  |  |
| *P. ful* | 0.217 | 0.217 | 0.222 | 0.003 | 0.003 | 0.002 |  |  |  |  |  |  |  |  |  |  |  |  |  |  |  |  |
| *L. cul* | 0.192 | 0.191 | 0.084 | 0.186 | 0.186 | 0.186 | 0.186 |  |  |  |  |  |  |  |  |  |  |  |  |  |  |  |
| *L. pub* | 0.175 | 0.173 | 0.106 | 0.160 | 0.160 | 0.160 | 0.160 | 0.075 |  |  |  |  |  |  |  |  |  |  |  |  |  |  |
| *L. ven* | 0.169 | 0.169 | 0.101 | 0.154 | 0.154 | 0.154 | 0.155 | 0.071 | 0.014 |  |  |  |  |  |  |  |  |  |  |  |  |  |
| *L. pal* | 0.221 | 0.220 | 0.128 | 0.201 | 0.202 | 0.201 | 0.202 | 0.119 | 0.067 | 0.058 |  |  |  |  |  |  |  |  |  |  |  |  |
| *L. jap* | 0.169 | 0.169 | 0.102 | 0.154 | 0.155 | 0.154 | 0.155 | 0.071 | 0.015 | 0.002 | 0.059 |  |  |  |  |  |  |  |  |  |  |  |
| *L. och* | 0.169 | 0.169 | 0.101 | 0.154 | 0.154 | 0.154 | 0.155 | 0.071 | 0.014 | 0.001 | 0.058 | 0.002 |  |  |  |  |  |  |  |  |  |  |
| *L. dav* | 0.216 | 0.216 | 0.128 | 0.196 | 0.197 | 0.196 | 0.197 | 0.119 | 0.063 | 0.054 | 0.008 | 0.054 | 0.054 |  |  |  |  |  |  |  |  |  |
| *L. lit* | 0.170 | 0.169 | 0.102 | 0.155 | 0.155 | 0.154 | 0.155 | 0.071 | 0.015 | 0.003 | 0.059 | 0.003 | 0.003 | 0.055 |  |  |  |  |  |  |  |  |
| *L. inc* | 0.226 | 0.225 | 0.132 | 0.204 | 0.204 | 0.204 | 0.205 | 0.122 | 0.073 | 0.070 | 0.013 | 0.070 | 0.070 | 0.019 | 0.070 |  |  |  |  |  |  |  |
| *L. gra* | 0.222 | 0.221 | 0.129 | 0.202 | 0.202 | 0.202 | 0.203 | 0.119 | 0.068 | 0.058 | 0.002 | 0.059 | 0.058 | 0.008 | 0.059 | 0.014 |  |  |  |  |  |  |
| *L. tin* | 0.182 | 0.181 | 0.106 | 0.153 | 0.153 | 0.152 | 0.153 | 0.076 | 0.048 | 0.042 | 0.098 | 0.043 | 0.042 | 0.094 | 0.043 | 0.101 | 0.099 |  |  |  |  |  |
| *L. cly* | 0.193 | 0.192 | 0.206 | 0.212 | 0.212 | 0.212 | 0.212 | 0.193 | 0.169 | 0.165 | 0.106 | 0.166 | 0.165 | 0.108 | 0.166 | 0.112 | 0.107 | 0.176 |  |  |  |  |
| *L. sat* | 0.209 | 0.209 | 0.223 | 0.241 | 0.240 | 0.240 | 0.241 | 0.213 | 0.202 | 0.200 | 0.138 | 0.200 | 0.200 | 0.140 | 0.200 | 0.142 | 0.139 | 0.193 | 0.098 |  |  |  |
| *L. odo* | 0.184 | 0.184 | 0.109 | 0.156 | 0.156 | 0.155 | 0.156 | 0.079 | 0.051 | 0.046 | 0.102 | 0.046 | 0.046 | 0.098 | 0.047 | 0.105 | 0.102 | 0.029 | 0.180 | 0.190 |  |  |
| *C. ari* | 0.246 | 0.246 | 0.087 | 0.247 | 0.247 | 0.246 | 0.247 | 0.110 | 0.132 | 0.128 | 0.156 | 0.129 | 0.128 | 0.157 | 0.128 | 0.159 | 0.157 | 0.132 | 0.229 | 0.248 | 0.136 |  |
| The numbers in the table represent average pairwise sequence distance of chloroplast genome sequences. Analyses were conducted using the Kimura 2-parameter model. The full name of twenty-two species: *V. sep*, *V. sepium*; *V. sat*, *V. sativa*; *V. fab*, *V. faba*; *P. aby, P.* *abyssinicum; P. sat*, *P. sativum*; *P. sat*^sub^, *P. sativum* subsp. elatius; *P. ful, P. fulvum*; *L. cul*, *L. culinaris*; *L. pub, L. pubescens; L. ven, L. venosus*; *L. pal*, *L. palustris*; *L. jap, L. japonicus*; *L. och, L. ochroleucus*; *L. dav*, *L. davidii*; *L. lit, L.* *littoralis; L. inc, L.* *inconspicuus; L. gra*, *L. graminifolius*; *L. tin*, *L. tingitanus*; *L. cly,* *L. clymenum; L. sat, L.sativus; L. odo, L.odoratus; C. ari*, *C. arietinum*. | | | | | | | | | | | | | | | | | | | | | | |
